# Analysis of the Tolerance to DNA Alkylating Damage in *MEC1* and *RAD53* Checkpoint Mutants of *Saccharomyces cerevisiae*

**DOI:** 10.1371/journal.pone.0081108

**Published:** 2013-11-19

**Authors:** Alfonso Gallego-Sánchez, Sandra Ufano, Sonia Andrés, Avelino Bueno

**Affiliations:** Instituto de Biología Molecular y Celular del Cáncer, Departamento de Microbiología y Genética, Universidad de Salamanca/CSIC, Salamanca, Spain; Texas A&M University, United States of America

## Abstract

Checkpoint response, tolerance and repair are three major pathways that eukaryotic cells evolved independently to maintain genome stability and integrity. Here, we studied the sensitivity to DNA damage in checkpoint-deficient budding yeast cells and found that checkpoint kinases Mec1 and Rad53 may modulate the balance between error-free and error-prone branches of the tolerance pathway. We have consistently observed that mutation of the *RAD53* counterbalances error-free and error-prone branches upon exposure of cells to DNA damage induced either by MMS alkylation or by UV-radiation. We have also found that the potential Mec1/Rad53 balance modulation is independent from Rad6/Rad18-mediated PCNA ubiquitylation, as *mec1Δ* or *rad53Δ* mutants show no defects in the modification of the sliding clamp, therefore, we infer that it is likely exerted by acting on TLS polymerases and/or template switching targets.

## Introduction

The DNA damage tolerance mechanism allows DNA replication forks to progress through chemically altered, or damaged, template strands preventing irreversible fork collapse during S phase. The sequential ubiquitylation of PCNA plays a key role in the control of tolerance to DNA damage in eukaryotes. PCNA is monoubiquitylated at Lysine 164 to enhance the affinity of error-prone DNA polymerases which facilitate translesion synthesis (TLS) and is eventually polyubiquitylated to promote template switching, the error-free component of lesion bypass that involves sister-strand recombination (recently reviewed in [1]). Although TLS polymerases (in *Saccharomyces cerevisiae* DNA polymerases ζ and η) may be error-prone when copying undamaged templates, they can use damaged templates that block replicative DNA polymerases δ and ε. Therefore, TLS polymerases provide a mechanism (by replicating over lesions in damaged DNA templates) for the replisome to sustain fork progression (for a review, see [2]).

The balance between error-prone and error-free TLS mechanisms is different between different species of living organisms, or even in distinct types of cells within the same organism (for a review, see [2]). This distinctive regulation may reflect changes in TLS polymerase usage in different cells or in dealing with different kinds of DNA lesions [2], [3]. These changes in the relative balance suggest the existence of a modulatory mechanism of control. In the unicellular budding yeast, *S. cerevisiae*, it is thought that error-prone and error-free branches are unbalanced towards the latter, as reported for the replication of plasmids with a defined photoproduct [4], such that cells bypass DNA lesions without necessarily increasing the mutagenic rate.

The control of PCNA ubiquitylation is a solidly tested model in eukaryotes, and it is accepted that it evolved independently from the Mec1/ATM-Rad53/CHK2 checkpoint response. In fact, studies in *S. cerevisiae* and *Schizosaccharomyces pombe* on a potential mutual dependence of the ATR checkpoint signalling and DNA damage tolerance mechanisms strongly suggest that they are different responses to DNA damage [5], [6]. However, in *Xenopus* and human cells a regulatory role of the ATR-mediated checkpoint response or some of its components to the ubiquitylation of PCNA cannot be excluded [7]–[12]. In the present work we tested this independence hypothesis again by studying the relative importance of the error-prone and error-free branches of DNA damage tolerance in *S. cerevisiae* cells mutated in the checkpoint response. We tested it by exploring the sensitivity to the DNA alkylating chemical methyl methanesulfonate (MMS) in *MEC1* and *RAD53* checkpoint deficient yeast cells. Unexpectedly, we found that cells deleted for *MEC1* (ATR homolog) and/or *RAD53* (Chk2 homolog) kinases are still tolerant to low doses of MMS. Furthermore, we also found that tolerance in *MEC1* mutant cells, like in wild-type cells, depends more on the error-free branch of the pathway. However, we also observed that mutation of budding yeast *RAD53* checkpoint kinase counterbalances error-free and error-prone branches of the tolerance pathway to MMS-mediated DNA damage. Remarkably, the counterbalance caused by mutation of *RAD53* depends on *MEC1*, as tolerance to the alkylating chemical in *mec1 rad53* double mutants is unbalanced towards the error-free branch, like in wild-type or *mec1* single mutant cells.

## Results

### Analysis of DNA damage tolerance pathway in hypomorphic *rad53* mutants

We have previously described an hypomorphic mutant allele of the effector checkpoint kinase *RAD53*, *rad53Ha* [13]. The *rad53Ha* mutant allele encodes a version of the budding yeast DNA-damage effector checkpoint kinase tagged with three Ha epitopes at the C-terminus. *rad53Ha* mutant cells produce a highly unstable protein, thus dramatically reducing the cellular levels of the checkpoint kinase [13] (Ufano and Bueno, unpublished results). A direct (and highly unexpected) consequence of this radical reduction in the checkpoint kinase is that *rad53Ha* cells are resistant to the chronic presence of the alkylating chemical MMS [13]. Unexpected because *rad53* null mutants are hypersensitive to MMS. Additionally, *rad53Ha* has a mutator phenotype [13], so we were interested in understanding the tolerance mechanism that underlies *rad53Ha* resistance to MMS.

**Figure 1:**
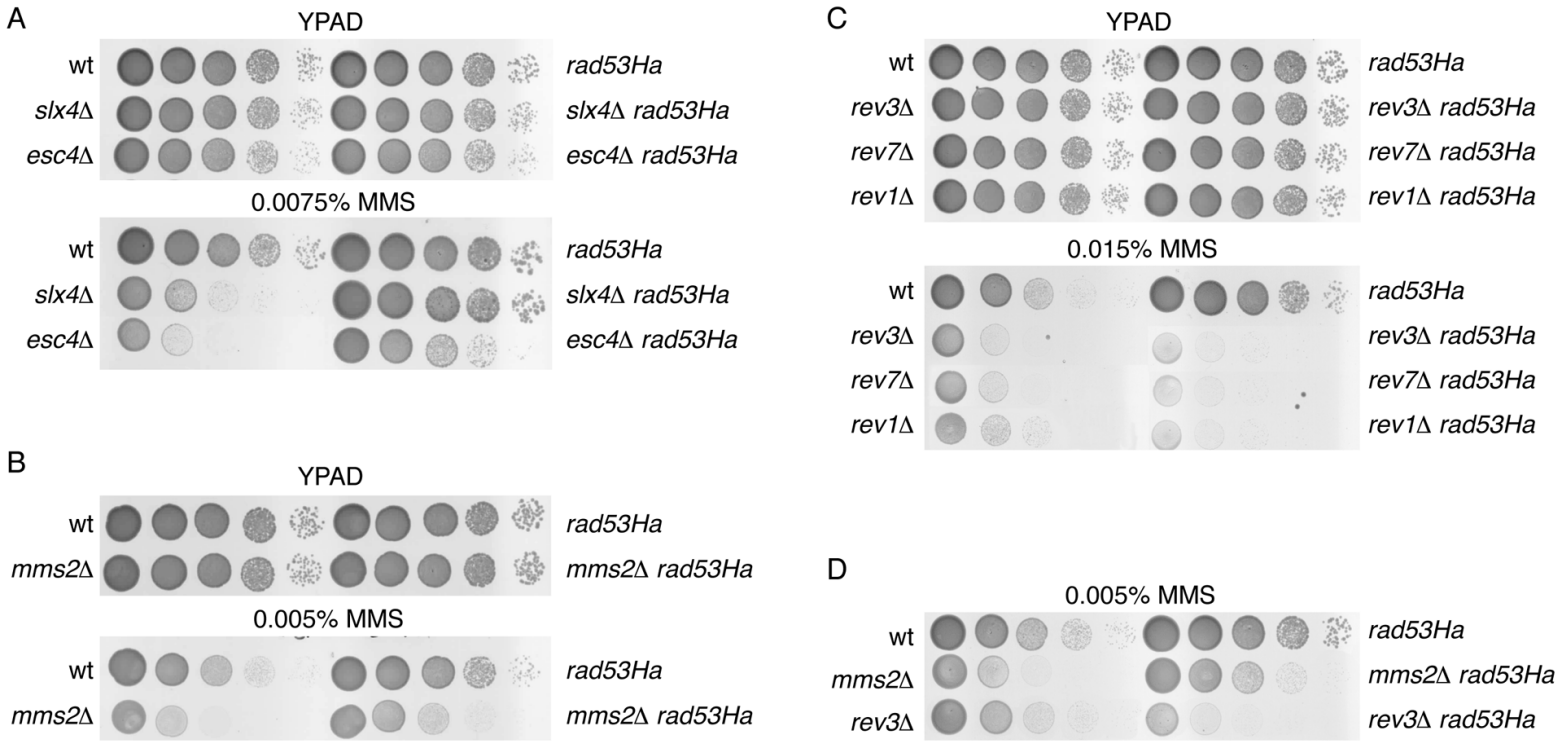
Analysis of DNA damage tolerance pathway in *rad53Ha* mutants. (A) Mutation of endonuclease *SLX4* or DNA repair protein *ESC4*/*RTT107* has little impact on MMS sensitivity of *rad53Ha* cells. Serial dilutions (ten-fold) of wild-type, *slx4Δ, esc4Δ*, *rad53Ha*, *slx4Δ rad53Ha* and *esc4Δ rad53Ha* cells were plated onto YPAD plates with and without MMS (as indicated). (B) Analysis of the effect of *MMS2* mutation on *rad53Ha* cells tolerance. As in (A) ten-fold serial dilutions of the indicated strains were plated onto YPAD plates with and without MMS. (C) Deletion of *REV1*, *REV*3 or *REV*7 suppresses resistance to MMS in *rad53Ha* cells. Ten-fold serial dilutions of the indicated strains are shown. (D) Relative balance of the error-prone and error-free tolerance pathways in *rad53Ha* cells. Serial dilutions (ten-fold) of wild-type, *rad53Ha*, *mms2Δ*, *rev3Δ*, *mms2Δ rad53Ha* and *rev3Δ rad53Ha* plated onto YPAD plates with 0.005% MMS or without the alkylating chemical. YPAD plates (YPD supplemented with 50 µg/ml Adenine) containing MMS were freshly made and used within 5–7 hours. Note: All the experiments shown in this work were repeated three times and with different clones of every mutant to ensure reproducibility.

We first compared the MMS sensitivity of a collection of gene deletions important for MMS survival [14] in both *RAD53* wild-type and *rad53Ha* mutant backgrounds. We found that, as described [15], deletion of *SLX4* or *ESC4*/*RTT107* rendered the cells hypersensitive to MMS. However, the *SLX4* or *ESC4*/*RTT107* mutations had little impact in a *rad53Ha* resistance to MMS (Fig. 1A). The Slx4 subunit of the heteromeric endonuclease Slx1-Slx4 and the BRCA1 C-terminal-domain protein Esc4 interact *in vivo* and are required for recovery from DNA damage during S-phase [15]-[19]. Previous studies have shown that *slx4Δ* is epistatic to mutations in *mms2* and *ubc13* error-free bypass genes regarding MMS hypersensitivity [15], [16]. However, *slx4Δ* is not epistatic to mutations in TLS polymerases [15], [16]. We therefore extended our genetic analysis of *rad53Ha* to *rev1Δ*, *rev3Δ*, *rev7Δ* and *mms2Δ* mutant cells (Fig. 1B, 1C and 1D). Rev1 is a deoxycytidyltransferase involved in the bypass of abasic sites in damaged DNA and it forms a complex with the subunits of DNA polymerase zeta (ζ) Rev3p and Rev7p, which are involved in error-prone lesion bypass (TLS DNA polymerases) [20], [21] (Figure S1). Mms2p is an ubiquitin-conjugating enzyme involved in error-free post-replication repair that forms a heteromeric complex with the ubiquitin-conjugating enzyme Ubc13p [22] (Figure S1). We carried out ten-fold serial dilutions and found that *mms2Δ rad53Ha* cells were less sensitive to the chronic presence of MMS than *mms2Δ* single mutants (Fig. 1B), suggesting that resistance to DNA damage in *rad53Ha* does not depends on the error-free branch of lesion bypass. In contrast, we also observed that when combined with *rad53Ha*, the *rev1Δ*, *rev3Δ* or *rev7Δ* double mutants were more sensitive to MMS than *rev1Δ*, *rev3Δ* or *rev7Δ* single mutants (Fig. 1C), indicating an absolute dependence of *rad53Ha* resistance on the error-prone TLS polymerases. Interestingly, in a *rad53Ha* genetic background the balance between the error-free and error-prone branches changed dramatically as compared to a wild-type (Fig. 1D), since *rev1Δ*, *rev3Δ* and *rev7Δ* prevailed over *mms2Δ*; this suggests that the effector checkpoint kinase may play a role in modulating post-replication repair (PRR). We next extended our analysis by studying cells carrying a different hypomorphic allele of *RAD53*, *rad53^S350A,G404V^*, that produced low levels of an active and stable form of the Rad53 protein (Ufano and Bueno, unpublished results). The MMS tolerance of *rad53^S350A,G404V^* cells was similarly dependent on TLS-polymerase ζ catalytic subunit Rev3 (Figure S2). These results are consistent with a modulatory role of Rad53p in the balance between the two branches of lesion bypass. Further support for this hypothesis comes from the analysis of *dot1Δ* mutant cells that tolerate MMS-mediated DNA damage in a Polζ/Rev1 dependent manner [23], [24]. Not surprisingly, *dot1Δ* mutant cells have defects in Rad53 activation [25]–[27].

### PCNA lysine 164 to arginine mutation prevents tolerance to MMS in *rad53Ha* cells

**Figure 2:**
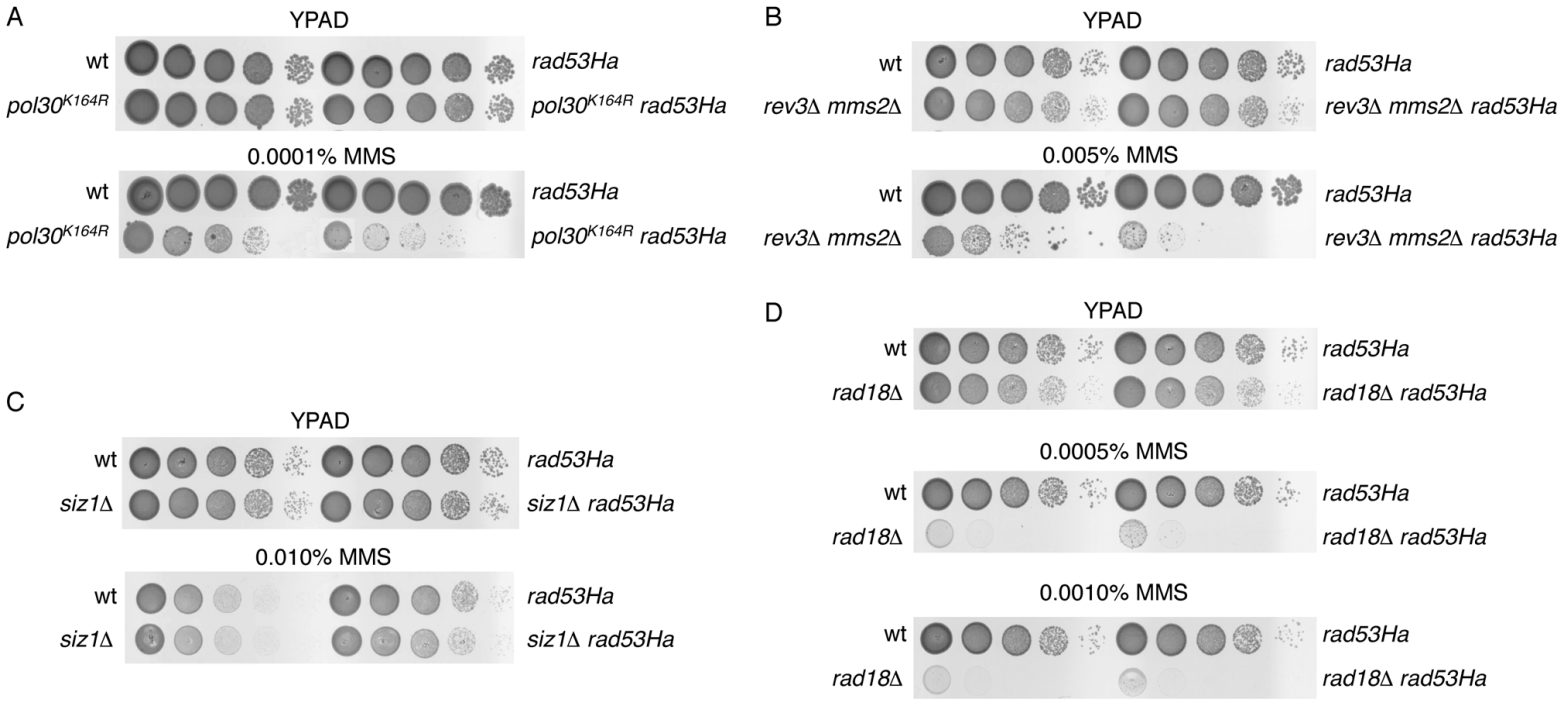
The Lysine 164 to Arginine mutation of *POL30* suppresses the MMS resistance of *rad53Ha*. (A) Serial dilutions (ten-fold) of wild-type, *rad53Ha*, *pol30^K164R^* and *pol30^K164R^ rad53Ha* cells were plated onto YPAD plates with and without MMS (as indicated). (B) Deletion of *MMS2* and *REV3* prevents tolerance to MMS in *rad53Ha* cells. Ten-fold serial dilutions on YPAD plates with or without MMS of the indicated strains are shown. (C) Resistance to MMS in *rad53Ha* cells is independent from PCNA SUMOylation. Ten-fold serial dilutions of wild-type, *rad53Ha*, *siz1Δ* and *siz1Δ rad53Ha* were plated onto YPAD plates with and without MMS. (D) Mutation of *RAD18* E3 ubiquitin ligase prevents tolerance to MMS-mediated DNA damage in *rad53Ha* mutants. Serial dilutions (ten-fold) of wild-type, *rad18Δ* and *rad18Δ rad53Ha* cells were plated on YPAD plates with and without MMS.

The control of the activation of error-prone and error-free mechanisms depends on covalent modifications of the sliding clamp PCNA [28], [29]. During S-phase, if damaged DNA is detected, the E2/E3-ubiquitin ligase complex Rad6/Rad18 monoubiquitylates PCNA on Lysine 164, enhancing the translesion synthesis pathway. Monoubiquitylated PCNA can be further polyubiquitylated at the same residue by a second E2/E3 complex, Mms2/Ubc13/Rad5, to activate the error-free branch (for a review, see [30]). We reasoned that if *rad53Ha* tolerance relies on PCNA ubiquitylation, a yeast strain carrying a *pol30^K164R^* allele should restrain the resistance to the chronic exposure to low levels of MMS, and indeed this was the case (Fig. 2A). Further support for this hypothesis came from the analysis of *mms2Δ rev3Δ rad53Ha* and *rad18Δ rad53Ha* mutants, in which both the error-prone and error-free branches were abrogated because TLS polymerases activity or PCNA ubiquitylation was prevented (Fig. 2B, 2D and Figure S3). Additionally, we tested the resistance of the *rad53Ha* mutant in a *siz1Δ* mutant background to show that the *rad53Ha* phenotype was independent of PCNA SUMOylation at the Lysine 164 residue (Fig. 2C). These observations indicate that PCNA ubiquitylation contributes to the *rad53Ha* resistance to MMS.

### *MEC1* and *RAD53*-checkpoint mutant cells are proficient in PCNA ubiquitylation

We next studied the status of PCNA ubiquitylation in *rad53Ha* and compared asynchronously growing and MMS-treated mutant (cells) with wild-type cells (Figure S4). We observed that *rad53Ha* mutant cells accumulated slightly more mono-, di-ubiquitylated, and SUMOylated PCNA than wild-type strains (Figure S4D). The observed increase in SUMO- and Ub-PCNA may be a consequence of a higher number of active replication forks. This hypothesis/idea is consistent with the deregulation of late origin firing previously reported that results in a greater number of fired origins and, in consequence, in more replication forks per cell [13]. A higher number of active replication forks explain the observed increase in SUMOylated- and ubiquitylated-PCNA in *rad53Ha* cells. In addition, these findings demonstrate that *rad53Ha* is proficient in PCNA ubiquitylation. Moreover, like other authors [5], we failed to observe any defect in PCNA ubiquitylation on Lysine 164 in *rad53Δ* and *mec1Δ* mutant cells (Figure S5). In particular, we observed that *rad53Δ* and *mec1Δ* mutant cells accumulated wild-type levels of ubiquitylated forms of PCNA upon exposure to different concentrations of MMS, indicating that they have no defects in PCNA ubiquitylation or deubiquitylation (as expected if checkpoint response and tolerance to DNA damage are independent pathways). Together, our data indicate that *rad53Ha* tolerance to MMS-induced DNA damage depends on PCNA ubiquitylation and strongly suggest that the control of this covalent modification of the sliding clamp is independent from the Mec1/Rad53 checkpoint cascade.

### Analysis of the DNA damage tolerance pathway in *mec1Δ*, *rad53Δ* and *chk1Δ* checkpoint kinase mutants

**Figure 3:**
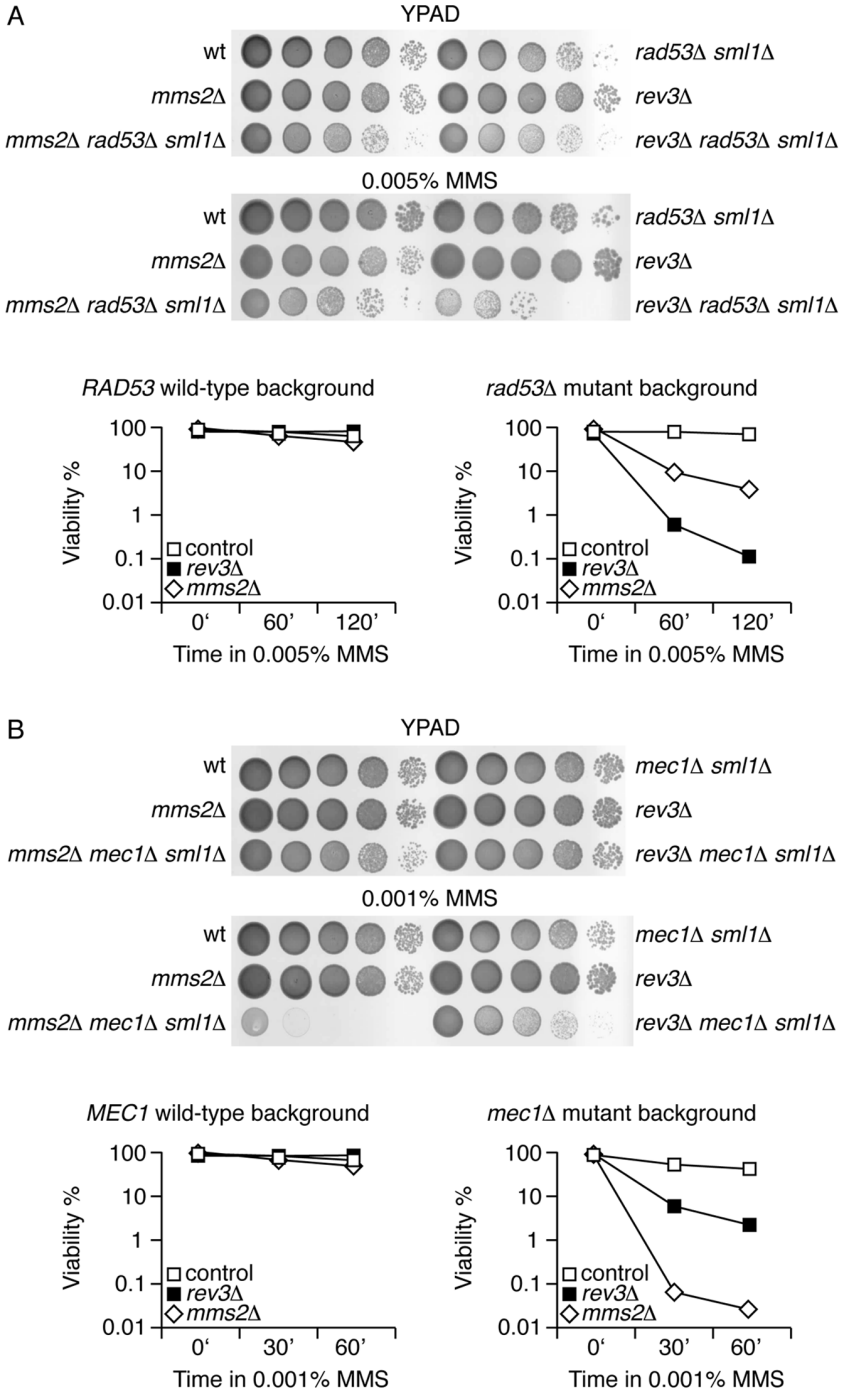
Analysis of the DNA damage tolerance pathway in *mec1* *Δ* and *rad53* *Δ* checkpoint kinase mutants. Serial dilutions (ten-fold) of indicated strains (see below) plated on YPAD plates with MMS and without the alkylating chemical. (A) wild-type, *rad53Δ sml1Δ*, *mms2Δ*, *rev3Δ*, *mms2Δ rad53Δ sml1Δ* and *rev3Δ rad53Δ sml1Δ*. (B) wild-type, *mec1Δ sml1Δ*, *mms2Δ*, *rev3Δ*, *mms2Δ mec1Δ sml1Δ* and *rev3Δ mec1Δ sml1Δ*. Viable plot graphs in (A) and (B) show the viability analysis of wild-type, *rad53Δ sml1Δ and mec1Δ sml1Δ* strains in *mms2Δ* or *rev3Δ* backgrounds (as indicated).

To further study the posible genetic relationship between checkpoint kinases and tolerance mechanism we analyzed the relative sensitivity to MMS of yeast strains lacking (checkpoint) signalling kinase Mec1, and effector kinases Rad53 or Chk1. If Mec1, Rad53 or Chk1 modulate somehow the relative importance of error-prone and error-free branches of PRR in yeast cells, their role might be easier to observe in cells lacking Rev3 or Mms2. Thus, we next characterized the MMS-sensitivity phenotype of *mec1Δ rev3Δ*, *mec1Δ mms2Δ*, *rad53Δ rev3Δ*, *rad53Δ mms2Δ*, *chk1Δ rev3Δ* and *chk1Δ mms2Δ* mutants (Fig. 3). Because of the hypersensitivity of *mec1Δ* and *rad53Δ* deletions to MMS, these experiments were carried out in the presence of low levels of the alkylating agent; in each case the concentration was estimated empirically, and we used the maximum concentration (of MMS) at which the single mutants showed tolerance. Consistent with the results obtained with *rad53Ha* and *rad53^S350A,G404V^*, deletion of *RAD53* changed the balance towards the error-prone branch of the tolerance pathway (Fig. 3A). This result supports the idea that Rad53 is negatively regulating TLS polymerases (Polζ/Rev3). Interestingly, and in contrast with *rad53Δ* mutant cells, *mec1Δ* deletion elicited a wild-type-like balance (Fig. 3B). However, the extreme hypersensitivity of *mec1Δ mms2Δ* indicated that the Mms2 (error-free)-dependent branch was more important for *mec1Δ* mutants than for the wild-type cells. Finally, no changes were observed in the tolerance balance in *chk1Δ* mutants (Figure S6). Together, these observations are consistent with the hypothesis/idea that Mec1 and Rad53 kinases may modulate the balance between the error-prone and error-free branches of tolerance to DNA damage.

### Analysis of the activation of the Mec1 kinase in *RAD53* mutant cells

**Figure 4:**
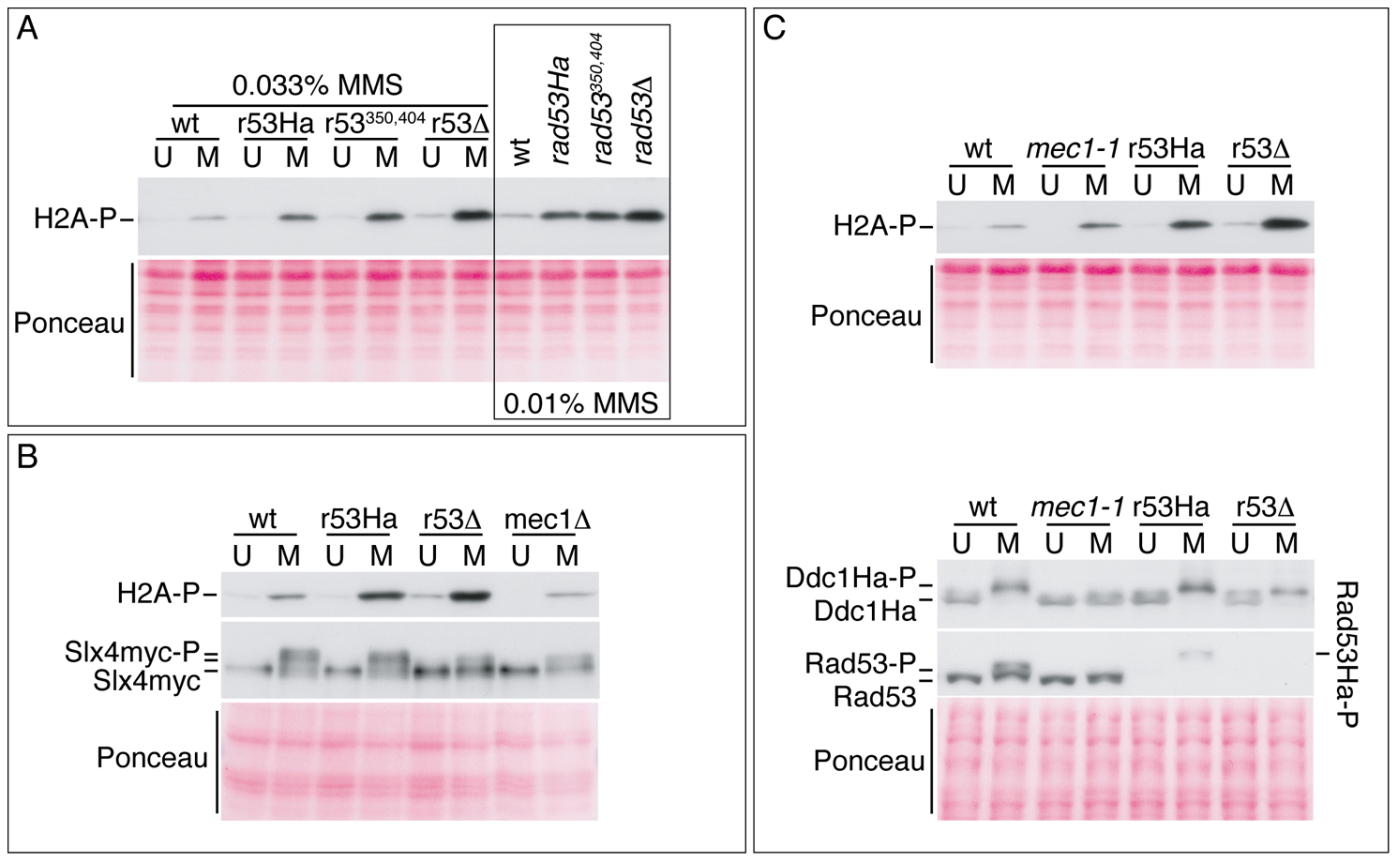
Phosphorylation of Mec1 and Mec1/Tel1 substrates in *rad53* mutant cells. TCA-extracted protein samples were taken from untreated cells (U) or 90 minutes MMS-treated cells (0.02% MMS except where indicated) (M), processed for Western blotting after SDS-PAGE in appropriate gels (from 8% to 12%), and probed with α-γH2AX (ab15083, Abcam), α-myc (M^.^5546, Sigma-Aldrich), α-Ha (12CA5, Sigma-Aldrich) or α-Rad53 (sc-6749, Santa Cruz Biotechnology Inc.) antibodies to detect histone H2A phosphorylated at S129, myc-tagged Slx4, Ha-tagged Ddc1 and Rad53, respectively. (A) γH2AX antibody immunoreactivity of whole cell extracts from wild-type and *rad53Ha* (r53Ha), *rad53^S350A,G404V^* (r53^350,404^) and *rad53Δ* (r53*Δ*) mutants untreated (U) or MMS-treated (M) as indicated. (B) (Upper panel) γH2AX antibody immunoreactivity and (middle panel) myc antibody immunoreactivity of whole cell extracts from *slx4-myc-*tagged cells, otherwise wild-type (wt), *rad53Ha* (r53Ha), *rad53Δ* (r53*Δ*) or *mec1Δ*, untreated (U) or MMS-treated (M). (C) Detection of γH2A (upper blot), Ddc1-Ha and Rad53 (lower blots) in whole cell extracts from *ddc1-Ha-*tagged cells, otherwise wild-type (wt), *mec1-1*, *rad53Ha* (r53Ha) or *rad53Δ* (r53*Δ*), untreated (U) or MMS-treated (M).

An alternative model in which only Mec1 would regulate the activity of TLS polymerases may explain the dependence of *rad53* mutants on the TLS pathway reported here. In this scenario the apparent regulatory role of Rad53 on TLS could be an observable but indirect effect of the potential hyper-activation of the Mec1 kinase in the absence of Rad53. Increased kinase activity should result in the hyper-phosphorylation of each Mec1 substrate, we therefore studied the phosphorylation of Mec1 (and Mec1/Tel1) substrates Ddc1, H2A and Slx4 in *rad53* mutants (Fig. 4). Previous work indicated that gamma-H2A (γH2A) and Slx4 are Mec1/Tel1 substrates [31]–[33] and that Ddc1 is an specific Mec1 substrate [34]. On the other hand, it has been shown that both Ddc1-Ha and Slx4-myc decrease their electrophoretic mobility when phosphorylated [33], [34] so that phosphoprotein isotypes can be detected by the band shift. In our experiments we found an increase in γH2A levels indicating that Mec1 is indeed hyper-activated, thus supporting Mec1 as the unique regulator for TLS activity (Fig. 4). However, contrary to the γH2A observation, we detected wild-type levels of phosphorylated Slx4 and Ddc1 in *rad53Ha* or *rad53Δ* cells suggesting that either Mec1 is not hyper-activated in these *rad53* mutants or, simply, that Ddc1 and Slx4 are fully phosphorylated in wild-type cells. We eventually tested the contribution of the Tel1 kinase (homolog of human ataxia-telangiectasia mutated, ATM, gene) to the H2A phosphorylation and found that Tel1 contribution to it is relatively minor (Figure S7). In summary, from these experiments we cannot fully discard any of the two above specified models. However, the net increase in phosphorylated H2A (γH2A) detected in *rad53* mutants strongly suggest that Mec1 is hyper-activated and favours the Mec1-dependent scenario/context.

### Analysis of the DNA damage tolerance pathway in *mec1Δ rad53Δ* checkpoint mutants

**Figure 5:**
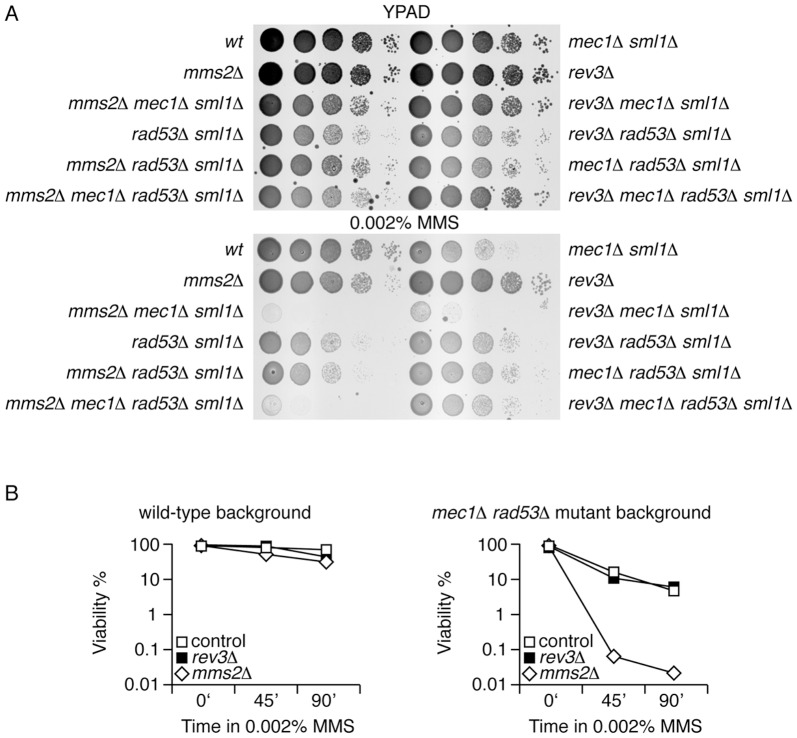
Analysis of the DNA damage tolerance pathway in *mec1* *Δ* *rad53* *Δ* double mutants. Serial dilutions (ten-fold) of indicated strains (see below) plated on YPAD plates with MMS or without the alkylating chemical. (A) wild-type, *mec1Δ sml1Δ*, *mms2Δ*, *rev3Δ*, *mec1Δ mms2Δ sml1Δ*, *mec1Δ rev3Δ sml1Δ*, *rad53Δ sml1Δ*, *rad53Δ rev3Δ sml1Δ*, *rad53Δ mms2Δ sml1Δ, mec1Δ rad53Δ sml1Δ, mec1Δ rad53Δ mms2Δ sml1Δ a*nd m*ec1Δ rad53Δ rev3Δ sml1Δ*. (B) Viability analysis of wild-type and *mec1Δ rad53Δ sml1Δ* strains in *mms2Δ* or *rev3Δ* backgrounds (as indicated).

To further investigate the relative role of Mec1 and Rad53 kinases in the balance of the error-prone and error-free tolerance branches, we next analyzed the sensitivity to MMS of *mec1Δ rad53Δ* cells in error-prone (*rev3Δ*) and error-free (*mms2Δ*) mutant backgrounds (Fig. 5). Similarly as described above for the single mutants analysis, the experiments were carried out in the presence of low levels of MMS as *mec1Δ rad53Δ* double mutants were expected to be highly sensitive to the alkylating agent. Again, the concentration of MMS used was the maximum at which the *mec1Δ rad53Δ* double mutant showed some degree of tolerance. Whereas the *sml1* mutation has no effect on MMS sensitivity (Figure S8), this epistasis analysis showed that *mec1Δ rad53Δ* double mutant cells were slightly more resistant to low concentrations of MMS (0.002%) than *mec1Δ* or *rad53Δ* single mutants. Interestingly, consistent with a Mec1-dependent control of the balance between the two branches of the tolerance pathway, the resistance of *mec1Δ rad53Δ* double mutant cells relied on the Mms2 (error-free) branch just like in *mec1Δ* single mutant or wild-type cells (Fig. 5A and 5B). We also tested the relative survival to UV radiation exposure of the strains assayed above and found a similar balance of the tolerance pathway to MMS-mediated damage in *rad53*, *mec1* and *rad53 mec1* mutants (Figure S9). Of particular interest is the observation that survival to UV radiation of *rad53* null mutant cells is based on the error-prone branch of the tolerance pathway (as for the MMS-treated cells) (Figure S9). Although these data do not rule out a direct effect of *RAD53* on the error-prone bypass, they indicate that regarding the balance of the branches of tolerance to MMS-induced DNA damage *MEC1* is epistatic to *RAD53*.

## Discussion

Here we have explored the consequences of mutating Mec1 or Rad53 in the tolerance pathway of *S.cerevisiae* cells when dealing with DNA damage induced by an alkylating chemical (MMS). Our studies indicate that mutation of budding yeast *RAD53* checkpoint kinase counterbalances error-free and error-prone branches of the tolerance pathway to DNA damage. These studies are based on experiments with the hypomorphic alleles *rad53Ha* and *rad53^S350A, G404V^* and also with *rad53Δ sml1Δ* deletion mutants of *S. cerevisiae* in which we observed an increased dependence of the tolerance to DNA damage on the error-prone branch (TLS-branch). These observations are in clear contrast with the balance observed in wild-type and *mec1Δ* mutant cells (both showing a predominant dependence on the error-free branch). We also show that the unbalance in *rad53* mutants is likely Mec1-dependent (as *MEC1 RAD53* double mutant cells display a wild-type like tolerance pattern). However, mutation of *RAD53* in a *mec1Δ sml1Δ* background increases the tolerance to MMS-induced DNA damage, suggesting a subtle inhibitory role of the Rad53-effector checkpoint kinase in the tolerance pathway. A possible explanation for these observations is that Rad53 would be partially required for error-free bypass and Mec1 would be partially required for TLS independently of each other. However, all these results are consistent also with a complex interplay between the S-phase checkpoint kinases and the error-prone and error-free branches of the tolerance pathway. Based on our results, we propose that the opposite effects of two key checkpoint kinases in yeast -the sensor kinase Mec1 and the effector kinase Rad53- on the regulation of translesion synthesis help to maintain the balance of the two branches of the DNA damage tolerance pathway. Further studies will be needed to confirm this hypothesis. Of particular interest in that sense will be to identify Mec1- and Rad53-specific substrates in the PRR (post-replication repair) pathways.

**Figure 6:**
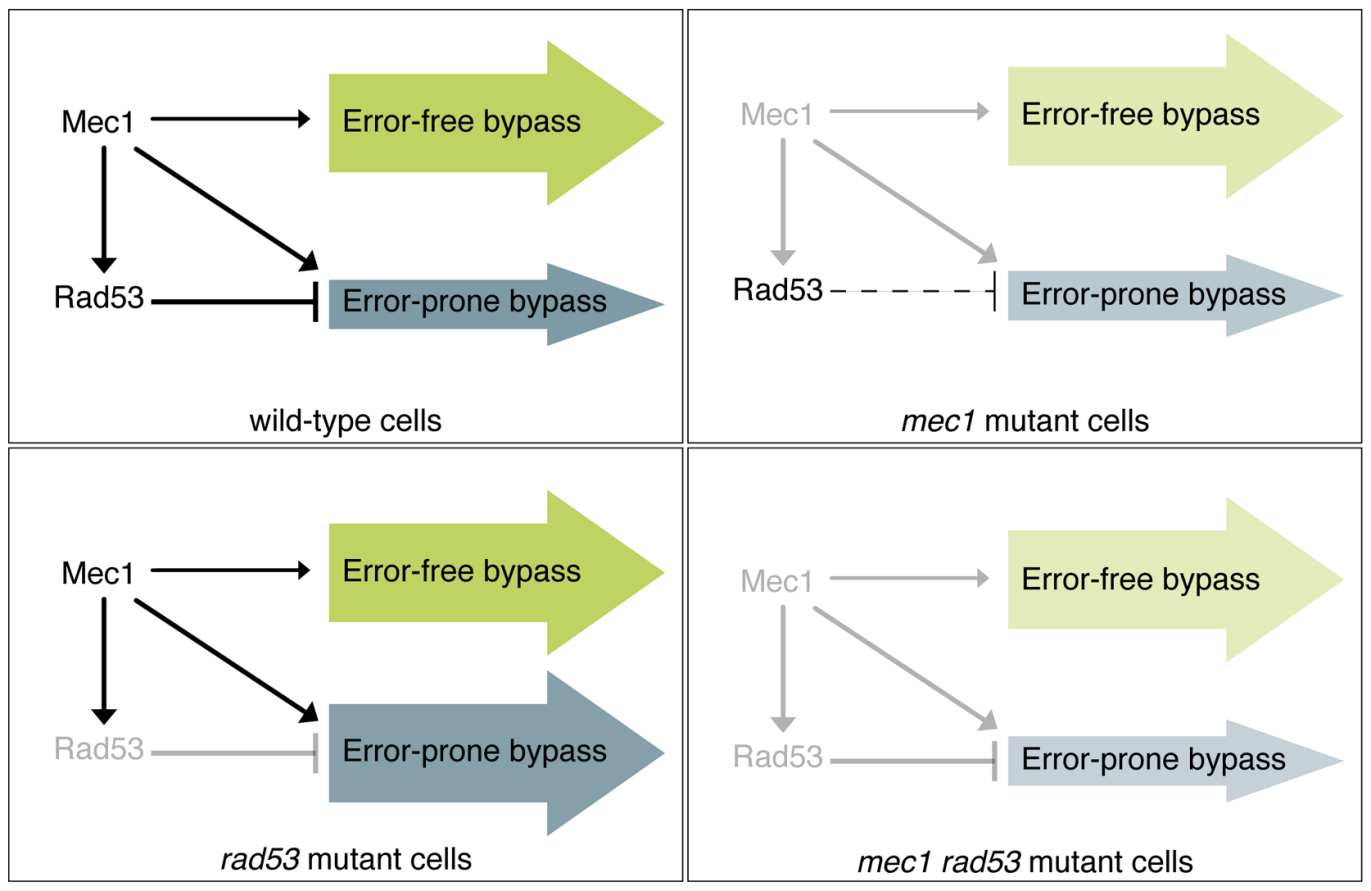
Model for Mec1p and Rad53p regulatory network in the tolerance pathway. In *S.cerevisiae*, a complex network links the S-phase checkpoint and the DNA damage tolerance pathway. We suggest that the opposite effects of two key S-phase checkpoint in yeast -the sensor kinase Mec1 and the effector kinase Rad53- help to maintain the balance of the two branches of the DNA damage-tolerance pathway. We propose that Rad53 would inhibit TLS DNA polymerase activity at least by limiting Rev1 foci formation. We also suggest that Mec1 would counterbalance Rad53 action on TLS polymerases, activating them either directly or through one of their positive effectors. At the same time Mec1 would also modulate Rad53 inhibitory effect through Rad53 activation. Additionally, Mec1 could activate the (error-free) template-switching branch of the tolerance pathway.

Analysis of fission and budding yeast cells has indicated that ATR/ATM-checkpoint signalling and tolerance are different responses to DNA damage that probably evolved independently [5], [6]. This is likely to reflect the independence of checkpoint kinases Mec1 and Rad53 from the control of PCNA ubiquitylation ([5], [6]; Figure S4 and S5). However, several lines of evidence support our hypothesis that checkpoint kinases modulate the balance between error-free and error-prone branches of the tolerance pathway regulating mutagenic translesion synthesis (see model in Fig. 6). Firstly, in budding yeast it has been reported that DNA polymerase ζ (TLS polymerase Rev3/Rev7) and Rev1 form a complex that associates with double-strand breaks (DSBs) in a Mec1-dependent manner [35]. Secondly, it has been shown that Ddc1p and Mec3p (9-1-1 components) interact *in vivo* with Rev3-subunit of Polζ [36]. Thirdly, deletion of *RAD9* in a *mms2Δ rev3Δ* double mutant increase the MMS-sensitivity (of *mms2Δ rev3Δ* mutants) to similar levels of *rad6Δ* or *rad18Δ* single mutants, suggesting that checkpoint proteins are involved in PRR [37]. 9-1-1 complex and Rad9 are respectively required for proficient activation of the checkpoint kinases Mec1 or Rad53. Fourthly, in fission yeast it has been shown that activation of the checkpoint plays a significant role in regulating the translesion synthesis polymerase DinB, pol-kappa which has no apparent budding yeast ortholog [38]. Finally, Rev1 is phosphorylated in wild-type *S.cerevisiae* cells following treatment with UV-light, zeocin and 4-NQO [39], [40]. Importantly, 4-NQO damage-induced Rev1 phosphorylation occurs in a *rad53Δ* strain but is lost in *mec1Δ* mutants [39], [40] suggesting that Mec1 not only regulates the association of Rev1 and polymerase ζ with chromatin in response to DSBs but also that it might contribute to enhancing the role of Rev1 in translesion synthesis.

Based on UV-damaged yeast cells analysis, it has been suggested that lesion bypass is regulated at the lesion site by the Mec1/Rad53 checkpoint–dependent mechanism of stabilization of stalled DNA replication forks [40], [41]. The evidence presented by Gangavarapu *et alii* [40], [41] is evocative of a role of Mec1 and Rad53 on the coordination of the tolerance pathway to DNA damage. In this regard, it has been shown that TLS polymerases remain functional in the absence of Mec1 and Rad53 [40]. On the other hand, however, it has been suggested that TLS activity may be modulated by Mec1 and Rad53 checkpoint kinases [40], [41]. In fact, as mentioned above, potentially direct Mec1 phosphorylation of Rev1 may contribute to increasing the proficiency of Polζ in lesion bypass [40]. Still, Rad53 is not required for TLS activity [40]. Even so, this latter evidence is not inconsistent with a negative modulator role of the Rad53 effector kinase on the Rev1-Rev3-Rev7-TLS pathway (as the one suggested here). On this point, we have recently shown that Rad53 modulates the tolerance pathway by down-regulating the abundance of chromatin-associated Rev1 foci in response to MMS [24].

In summary, the results presented here indicate that Rad53 mutant cells harbour hyperactivated Mec1 as well as they depend more on the TLS-pathway to tolerate the DNA damage induced either by MMS alkylation or by UV-radiation. Additionally, we have reported earlier that *rad53Ha* cells exhibit an enhanced rate of mutagenesis and have increased numbers of Rev1 foci bound to chromatin [13], [24]. Together, these lines of evidence suggest the implication of the Mec1 and Rad53 checkpoint kinases in the regulation of downstream effectors of the tolerance pathway.

## Materials and Methods

General methods of Molecular and Cellular Biology were used as described [42]. All the budding yeast used in our studies belong to a RAD5 W303 genetic background (Table S1). Yeast strains were grown in rich YPA medium (1% yeast extract, 2% peptone, supplemented with 50 µg/ml adenine) containing 2% glucose.

### MMS and Drugs Sensitivity Assays

Exponentially growing or stationary cells were counted and serially diluted in YPA media. Tenfold dilutions of equal numbers of cells were used. 10 µl of each dilution were spotted onto YPAD (2% glucose) plates (always supplemented with 50 µg/ml adenine), or YPAD plates containing different concentrations of MMS (as indicated), incubated at 25°C and scanned. MMS plates were always freshly made.

### MMS Survival Assays

Cells were grown to logarithmic phase in YPAD media at 25°C. Cultured cells were washed in fresh media, sonicated to disperse clumpy cells and resuspended to a density of 2×10^6^ cells per ml. Under these conditions cells were treated with MMS (as indicated in each experiment), washed, diluted and spread onto YPAD plates. Plates were incubated at 25°C for colony counting and cell viability determinations.

### UV Sensitivity Assays

Cells were grown to logarithmic phase in YPAD media at 25°C. Cultured cells were washed in fresh media, sonicated to disperse clumpy cells and resuspended to a density of 2×10^6^ cells per ml. Tenfold dilutions of these cells were spotted onto YPAD (2% glucose) plates (always supplemented with 50 µg/ml adenine). The plates were UV irradiated (as indicated) and incubated in the dark for 3 to 4 days at 25°C and scanned.

### Gene Deletions

Deletions of individual genes were made using a single-step PCR-based integration system [43] and confirmed by PCR (the deletions used in this work were generated in our lab unless otherwise noted: see Table S1). The selection markers used were *KANMX6*, which allows selection with Geneticin or *HphMX4*, which allows selection with hygromicin. We used also *ADE2*, *LEU2*, *HIS3* and *TRP1* markers (as indicated in Table S1). *rad53Ha* mutant strains were made as described [13]. In some cases single mutant alleles were crossed to make strains for this study (after selection of *MATa* segregants). The *rad53^S350A,G404V^* allele was constructed by gene replacement of the endogenous locus by a *RAD53*-containing DNA fragment in which T1048G and G1211T mutations had been introduced in the ORF of the gene by using PfuTurbo® DNA polymerase (Stratagene) for site-directed mutagenesis with appropriate oligonucleotides. All strains were confirmed either by PCR (deletions) or by PCR and sequencing (those involving *rad53Ha* and *rad53^S350A,G404V^)* and (in all cases) phenotype testing.

### Immunoprecipitation, Western Blot Analysis and Antibodies

**Protein Extract Preparation for Western Analysis.** TCA cell extracts were prepared and analyzed as described previously [13], [44]. SDS-PAGE gels at 15%, 12%, 10% and 7.5% were used for detection of histone H2B, PCNA (12% and 10%) and Rad53, respectively. 

**Protein Extract Preparation for Immunoprecipitations.** Soluble protein extracts were prepared basically as described previously [42], [45]. Cells were collected, washed, and broken in HB2T buffer using glass beads. The HB2T buffer contained 60 mM β-glycerophosphate, 15 mM *p*-nitrophenylphosphate, 25 mM 4-morpholinepropanesulfonic acid (pH 7.2), 15 mM MgCl2, 15 mM EGTA, 1 mM dithiothreitol, 0.1 mM sodium orthovanadate, 2% Triton X-100, 1 mM phenylmethylsulfonyl fluoride, and 20 mg/ml leupeptin and aprotinin. The glass beads were washed with 500 ml of HB2T, and the supernatant was recovered. Protein concentrations were measured using the BCA assay kit (Pierce). Protein samples were immunoprecipitated with affinity-purified PCNA antibody, processed for Western blotting after SDS-PAGE in 12% gels and probed with α-Ubiquitin (sc-8017, Santa Cruz Biotechnology Inc.) and α-PCNA antibodies. For Western blots, 40–80 µg of total protein extracts from each sample were blotted onto nitrocellulose, and proteins were detected using a previously characterized anti-PCNA affinity-purified polyclonal antibody (1:1500) [46]. We also used Rad53 affinity-purified goat polyclonal antibody from Santa Cruz Biotechnology (used as indicated by the supplier), as well as the 12CA5 monoclonal antibody (Roche Molecular Biochemicals; 1:500), or the anti-Myc monoclonal antibody (Sigma-Aldrich; 1:3000).

## Supporting Information

Figure S1 **Model of the regulation of PCNA covalent modifications of Lysine 164 in response to DNA damage during S-phase.**
(JPG)

Figure S2 ***rad53^S350A,G404V^*** **tolerance to MMS depends on the catalytic subunit of DNA polymerase ζ Rev3.** (A) Serial dilutions (ten-fold) of wild-type, *rad53^S350A,G404V^*, *mms2Δ*, *rev3Δ*, *mms2Δ rad53^S350A,G404V^* and *rev3Δ rad53^S350A,G404V^* were plated on YPAD plates with 0.015% MMS or without the alkylating chemical. Note: *rad53^S350A,G404V^* mutant cells are slightly sensitive to HU (Ufano and Bueno, unpublished results) and wild-type-like regarding sensitivity to MMS. *rad53^S350A,G404V^* mutant cells produced low levels of an active and stable form of the Rad53 protein (Ufano and Bueno, unpublished results). (B) Viability analysis of wild-type, *mms2Δ*, *rev3Δ (left* plot), *rad53^S350A,G404V^*, *mms2Δ rad53^S350A,G404V^* and *rev3Δ rad53^S350A,G404V^* (right plot) strains. Exponentially growing cultures of the indicated strains were exposed to 0.015% MMS and tested for colony formation. Plot graphs of the resulting viability test are shown.
(JPG)

Figure S3 **Relative tolerance of** ***rad18*** *Δ* **and** ***mms2*** *Δ* ***rev3*** *Δ* **mutant cells in** ***RAD53*** **and** ***rad53Ha*** **backgrounds.** Serial dilutions (ten-fold) of wild-type, *rad18Δ, mms2Δ rev3Δ*, *rad53Ha*, *rad18Δ rad53Ha* and *mms2Δ rev3Δ rad53Ha* were plated on YPAD plates with 0.0001% MMS, 0.01% MMS or without the alkylating chemical incubated at 25°C during 60 hours. NOTE: This result suggests that E3 ubiquitin ligase Rad18 may have additional roles in PRR, such as regulating the activity of an alternative bypass pathway, like Polη, or in checkpoint response activation.
(JPG)

Figure S4 **Increased levels of PCNA ubiquitylation and SUMOylation in** ***rad53Ha*** **mutants.** (A) Immunoblot analysis of cell extracts from wild-type, *rad53Ha*, *rad18Δ*, *pol30^K164R^* and *mms2Δ* strains, untreated or MMS-treated (as indicated), is shown. TCA-extracted protein samples were taken after treatments, processed for Western blotting after SDS-PAGE in 10% gels, and probed with affinity-purified PCNA antibody. (B) Immunoblot analysis of cell extracts from wild-type, *rad53Ha*, *rad18Δ*, *rad18Δ rad53Ha*, *siz1Δ* and *siz1Δ rad53Ha* strains, untreated or MMS-treated (as indicated), is shown. TCA-extracted protein samples were taken after treatments, processed for Western blotting after SDS-PAGE in 12% gels, and probed with affinity-purified PCNA antibody. (C) Left panels, immunoblot analysis of cell extracts from wild-type (wt) and *rad53Ha* (53Ha) strains growing asynchronously (Asyn), treated 90 minutes with 0.02% MMS (MMS) or blocked in G1 (180 minutes in α-factor) is shown. Samples were processed as in A. Right panels, immunoblot analysis of PCNA immunoprecipitates from wild-type (wt) and *rad53Ha* (53Ha) strains growing asynchronously (Asyn), treated 90 minutes with 0.02% MMS (MMS) or blocked in G1 (180 minutes in α-factor) is shown. Protein samples were immunoprecipitated with affinity-purified PCNA antibody, processed for Western blotting after SDS-PAGE in 12% gels and probed with α-Ubiquitin (sc-8017, Santa Cruz Biotechnology Inc.) and α-PCNA antibodies. (D) A plot of the quantitation of PCNA ubiquitylation and SUMOylation in wild-type and *rad53Ha* cells from three independent experiments is shown (from samples of cells treated 1 hour with 0.020% MMS). In each case the wild-type samples served as reference (100%).
(JPG)

Figure S5 ***mec1▵*** **or** ***rad53▵*** **mutant cells show no defects in PCNA ubiquitylation.** Immunoblot analysis of cell extracts from wild-type, *mec1Δ sml1Δ*, *rad53Δ sml1Δ* and *rad18Δ* strains, untreated or MMS-treated (as indicated), is shown. TCA-extracted protein samples were taken after treatments, processed for Western blotting after SDS-PAGE in 10% gels, and probed with affinity-purified PCNA antibody. Samples from α-factor blocked wild-type cells and *rad18Δ* cells were used as negative controls (as PCNA cannot be ubiquitylated in G1 or in the absence of Rad18). A plot of the quantitation of PCNA ubiquitylation (Ub-PCNA) is shown.
(JPG)

Figure S6 **Analysis of the DNA damage tolerance pathway in a** ***chk1*** *Δ* **chekpoint kinase mutant.** Serial dilutions (ten-fold) of wild-type, *chk1Δ*, *mms2Δ*, *rev3Δ*, *mms2Δ chk1Δ* and *rev3Δ chk1Δ* strains plated on YPAD plates with MMS and without the alkylating chemical (as indicated).
(JPG)

Figure S7 **Phosphorylation of histone H2A in** ***tel1*** **mutant cells.** TCA-extracted protein samples of the indicated strans were taken from untreated cells (U) or 90 minutes MMS-treated cells (0.02% MMS except where indicated) (M), processed for Western blotting after SDS-PAGE in 13% gels and probed with α-γH2AX (ab15083, Abcam) to detect histone H2A phosphorylated at S129. A plot of the quantitation of H2A phosphorylation is shown.
(JPG)

Figure S8 **Analysis of the DNA damage tolerance pathway in** ***mms2Δ sml1Δ*** **and** ***rev3Δ sml1Δ*** **double mutants.** Serial dilutions (ten-fold) of indicated strains plated on YPAD plates with MMS or without the alkylating chemical. The wild-type, *mms2Δ*, *rev3Δ*, *mms2Δ sml1Δ* and *rev3Δ sml1Δ* strains were assayed to test whether the sml1 mutation has any (additive) effect on *mms2Δ* or *rev3Δ* mutations.
(JPG)

Figure S9 **Analysis of the DNA damage tolerance pathway in** ***mec1*** *Δ* ***rad53*** *Δ* **double mutants.** Serial dilutions (ten-fold) of indicated strains (see below) plated on YPAD plates and exposed to the indicated doses of UV radiation. wild-type, *mec1Δ*, *mms2Δ*, *rev3Δ*, *mec1Δ mms2Δ*, *mec1Δ rev3Δ*, *rad53Δ*, *rad53Δ rev3Δ*, *rad53Δ mms2Δ, mec1Δ rad53Δ, mec1Δ rad53Δ mms2Δ a*nd m*ec1Δ rad53Δ rev3Δ*. Note that all the strains used in this assay were *sml1Δ*.
(JPG)

Table S1 **List of strains used in this study.**
(PDF)
