# Supplementary figures and images for: Analysis of the Tolerance to DNA Alkylating Damage in MEC1 and RAD53 Checkpoint Mutants of Saccharomyces cerevisiae
Source: PLoS One. 2013 Nov 19;8(11):e81108. doi: 10.1371/journal.pone.0081108 (PMC3834268; doi:10.1371/journal.pone.0081108)

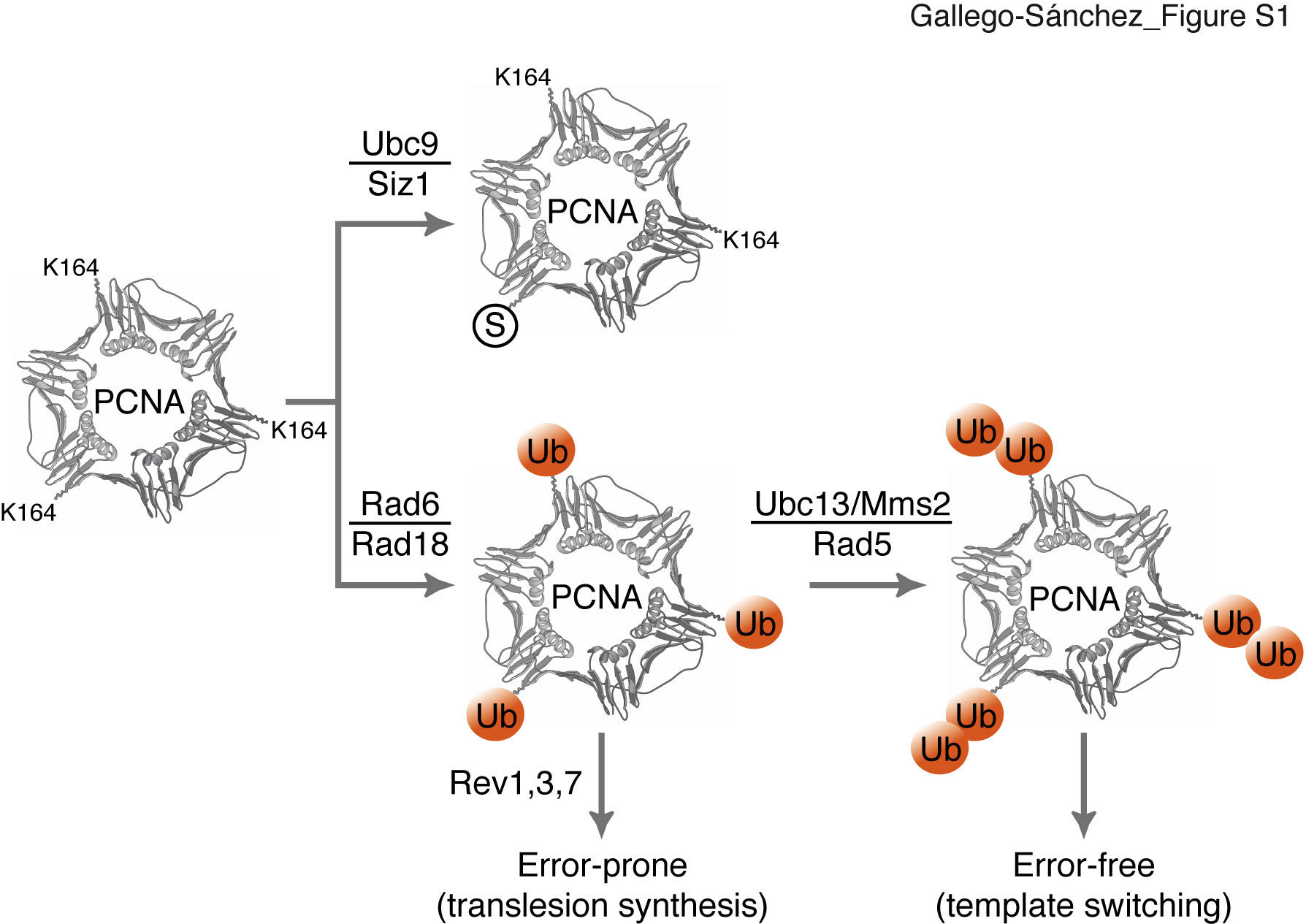

Supplement: Figure S1 — Model of the regulation of PCNA covalent modifications of Lysine 164 in response to DNA damage during S-phase. (JPG) [file pone.0081108.s001.jpg]

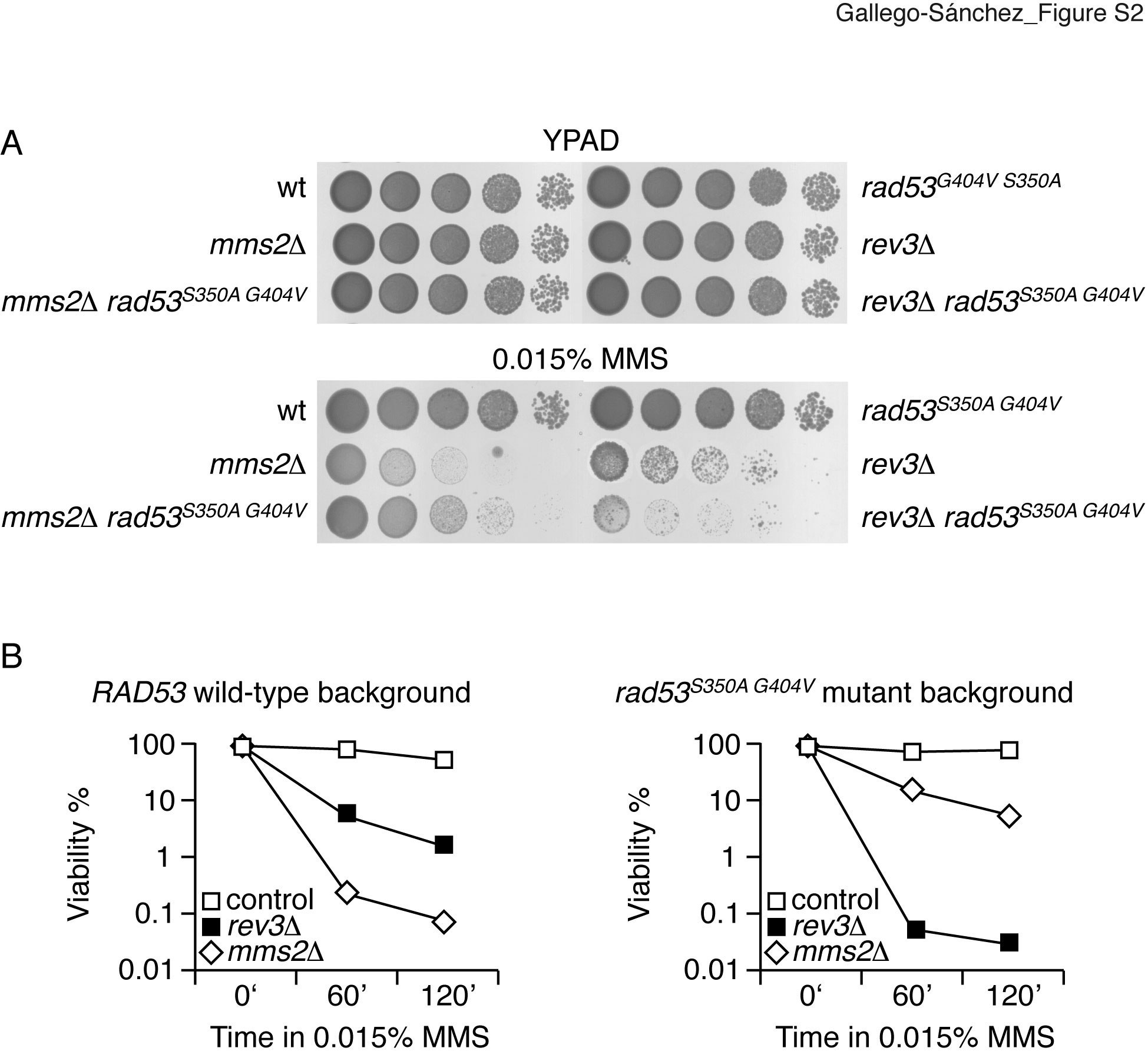

Supplement: Figure S2 — rad53S350A,G404V tolerance to MMS depends on the catalytic subunit of DNA polymerase ζ Rev3. (A) Serial dilutions (ten-fold) of wild-type, rad53S350A,G404V, mms2Δ, rev3Δ, mms2Δ rad53S350A,G404V and rev3Δ rad53S350A,G404V were plated on YPAD plates with 0.015% MMS or without the alkylating chemical. Note: rad53S350A,G404V mutant cells are slightly sensitive to HU (Ufano and Bueno, unpublished results) and wild-type-like regarding sensitivity to MMS. rad53S350A,G404V mutant cells produced low levels of an active and stable form of the Rad53 protein (Ufano and Bueno, unpublished results). (B) Viability analysis of wild-type, mms2Δ, rev3Δ (left plot), rad53S350A,G404V, mms2Δ rad53S350A,G404V and rev3Δ rad53S350A,G404V (right plot) strains. Exponentially growing cultures of the indicated strains were exposed to 0.015% MMS and tested for colony formation. Plot graphs of the resulting viability test are shown. (JPG) [file pone.0081108.s002.jpg]

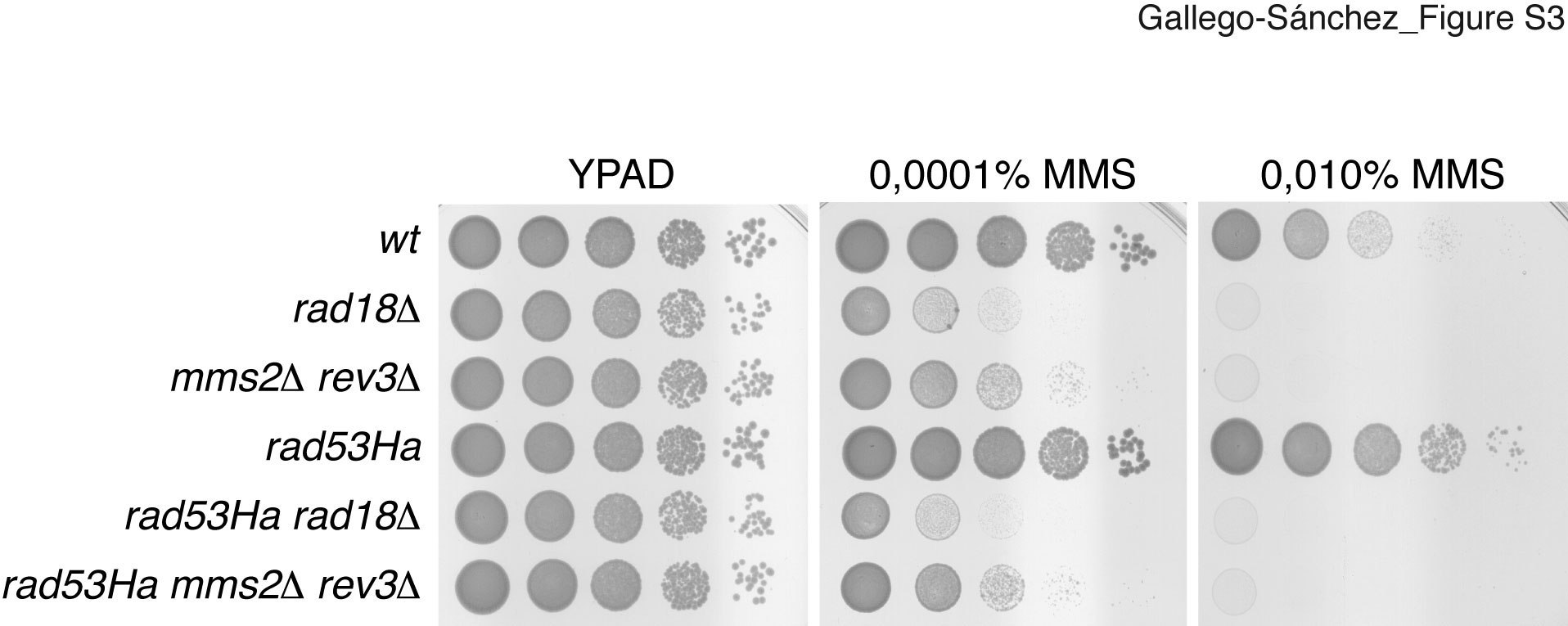

Supplement: Figure S3 — Relative tolerance of rad18 Δ and mms2 Δ rev3 Δ mutant cells in RAD53 and rad53Ha backgrounds. Serial dilutions (ten-fold) of wild-type, rad18Δ, mms2Δ rev3Δ, rad53Ha, rad18Δ rad53Ha and mms2Δ rev3Δ rad53Ha were plated on YPAD plates with 0.0001% MMS, 0.01% MMS or without the alkylating chemical incubated at 25°C during 60 hours. NOTE: This result suggests that E3 ubiquitin ligase Rad18 may have additional roles in PRR, such as regulating the activity of an alternative bypass pathway, like Polη, or in checkpoint response activation. (JPG) [file pone.0081108.s003.jpg]

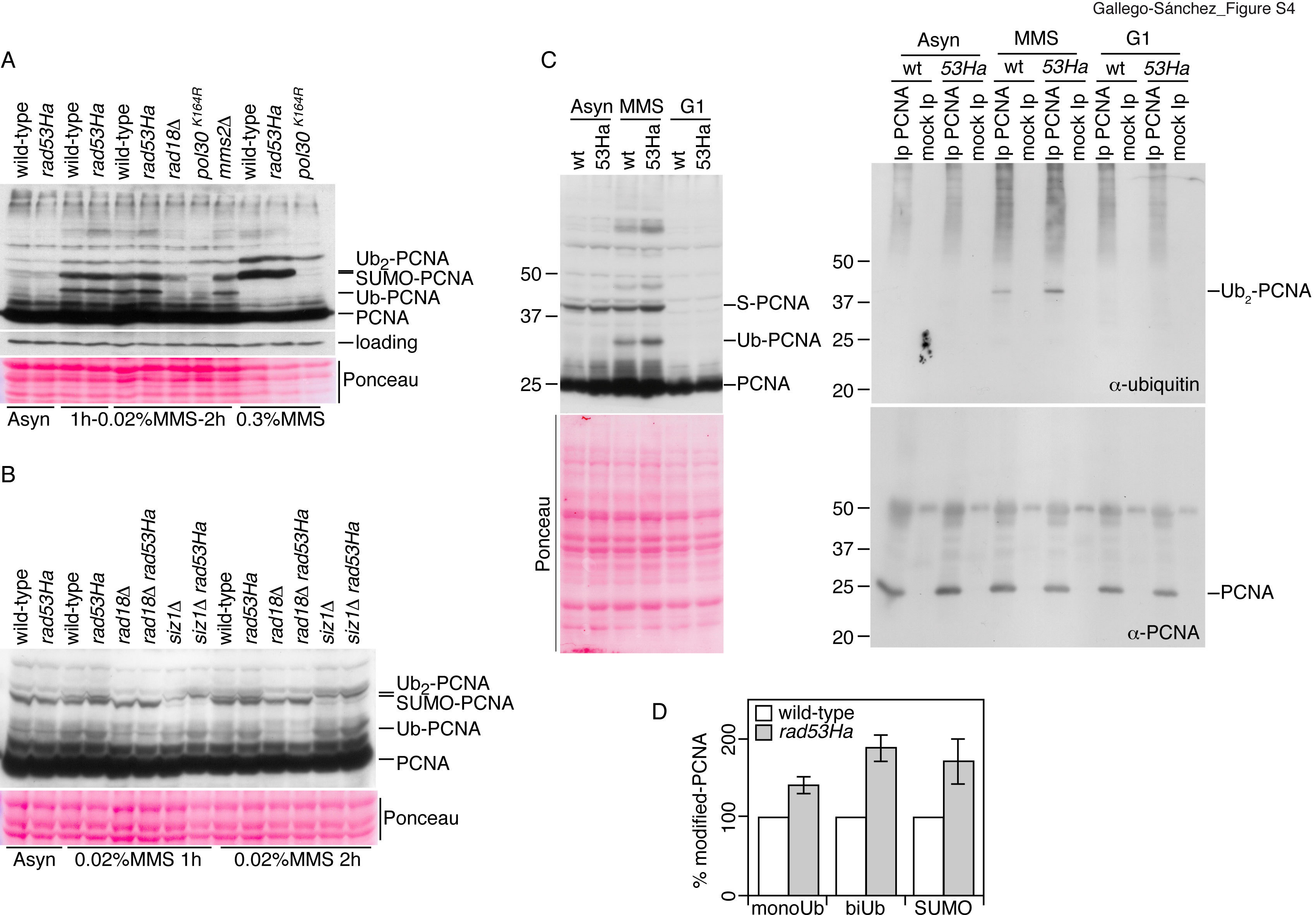

Supplement: Figure S4 — Increased levels of PCNA ubiquitylation and SUMOylation in rad53Ha mutants. (A) Immunoblot analysis of cell extracts from wild-type, rad53Ha, rad18Δ, pol30K164R and mms2Δ strains, untreated or MMS-treated (as indicated), is shown. TCA-extracted protein samples were taken after treatments, processed for Western blotting after SDS-PAGE in 10% gels, and probed with affinity-purified PCNA antibody. (B) Immunoblot analysis of cell extracts from wild-type, rad53Ha, rad18Δ, rad18Δ rad53Ha, siz1Δ and siz1Δ rad53Ha strains, untreated or MMS-treated (as indicated), is shown. TCA-extracted protein samples were taken after treatments, processed for Western blotting after SDS-PAGE in 12% gels, and probed with affinity-purified PCNA antibody. (C) Left panels, immunoblot analysis of cell extracts from wild-type (wt) and rad53Ha (53Ha) strains growing asynchronously (Asyn), treated 90 minutes with 0.02% MMS (MMS) or blocked in G1 (180 minutes in α-factor) is shown. Samples were processed as in A. Right panels, immunoblot analysis of PCNA immunoprecipitates from wild-type (wt) and rad53Ha (53Ha) strains growing asynchronously (Asyn), treated 90 minutes with 0.02% MMS (MMS) or blocked in G1 (180 minutes in α-factor) is shown. Protein samples were immunoprecipitated with affinity-purified PCNA antibody, processed for Western blotting after SDS-PAGE in 12% gels and probed with α-Ubiquitin (sc-8017, Santa Cruz Biotechnology Inc.) and α-PCNA antibodies. (D) A plot of the quantitation of PCNA ubiquitylation and SUMOylation in wild-type and rad53Ha cells from three independent experiments is shown (from samples of cells treated 1 hour with 0.020% MMS). In each case the wild-type samples served as reference (100%). (JPG) [file pone.0081108.s004.jpg]

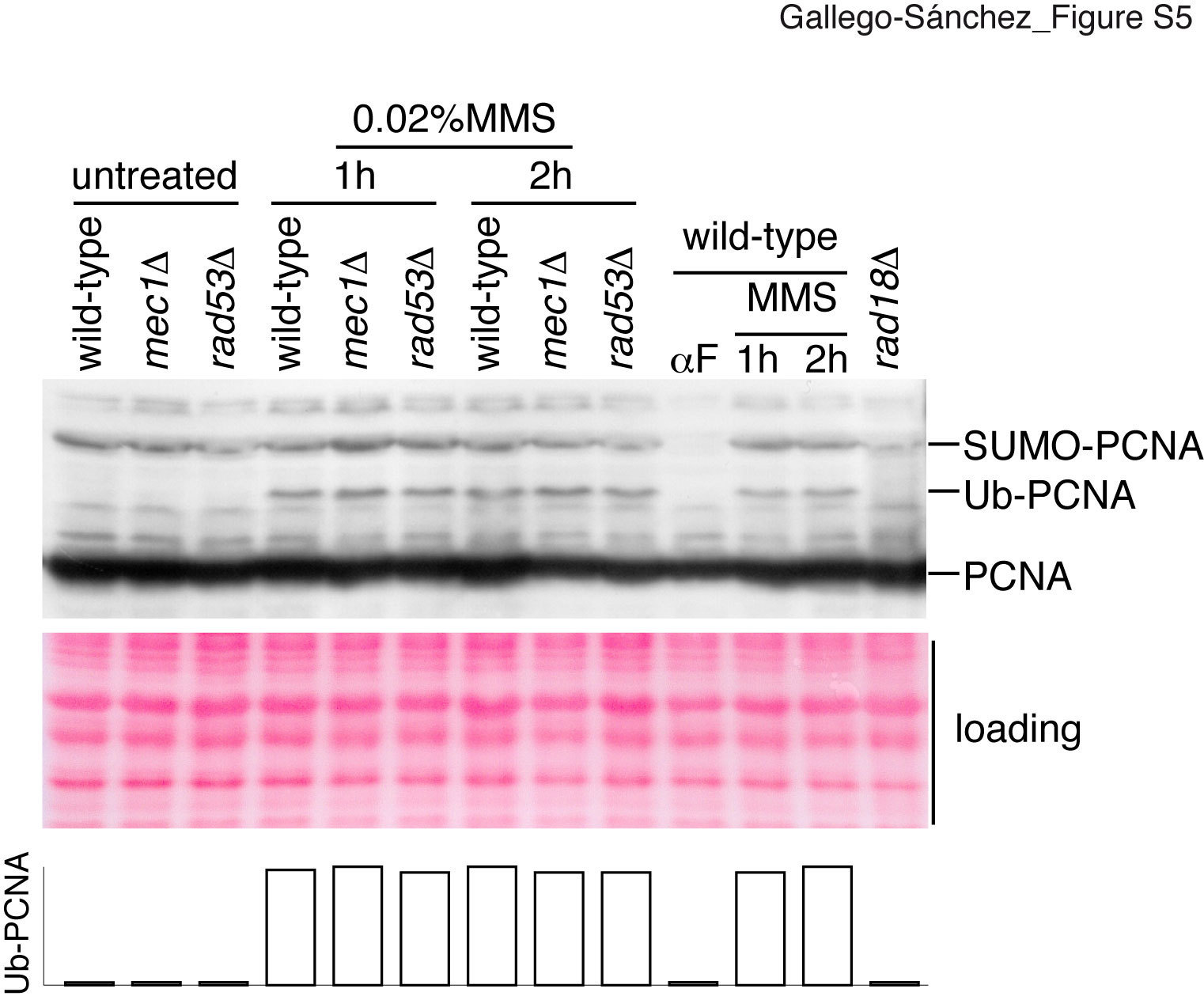

Supplement: Figure S5 — mec1▵ or rad53▵ mutant cells show no defects in PCNA ubiquitylation. Immunoblot analysis of cell extracts from wild-type, mec1Δ sml1Δ, rad53Δ sml1Δ and rad18Δ strains, untreated or MMS-treated (as indicated), is shown. TCA-extracted protein samples were taken after treatments, processed for Western blotting after SDS-PAGE in 10% gels, and probed with affinity-purified PCNA antibody. Samples from α-factor blocked wild-type cells and rad18Δ cells were used as negative controls (as PCNA cannot be ubiquitylated in G1 or in the absence of Rad18). A plot of the quantitation of PCNA ubiquitylation (Ub-PCNA) is shown. (JPG) [file pone.0081108.s005.jpg]

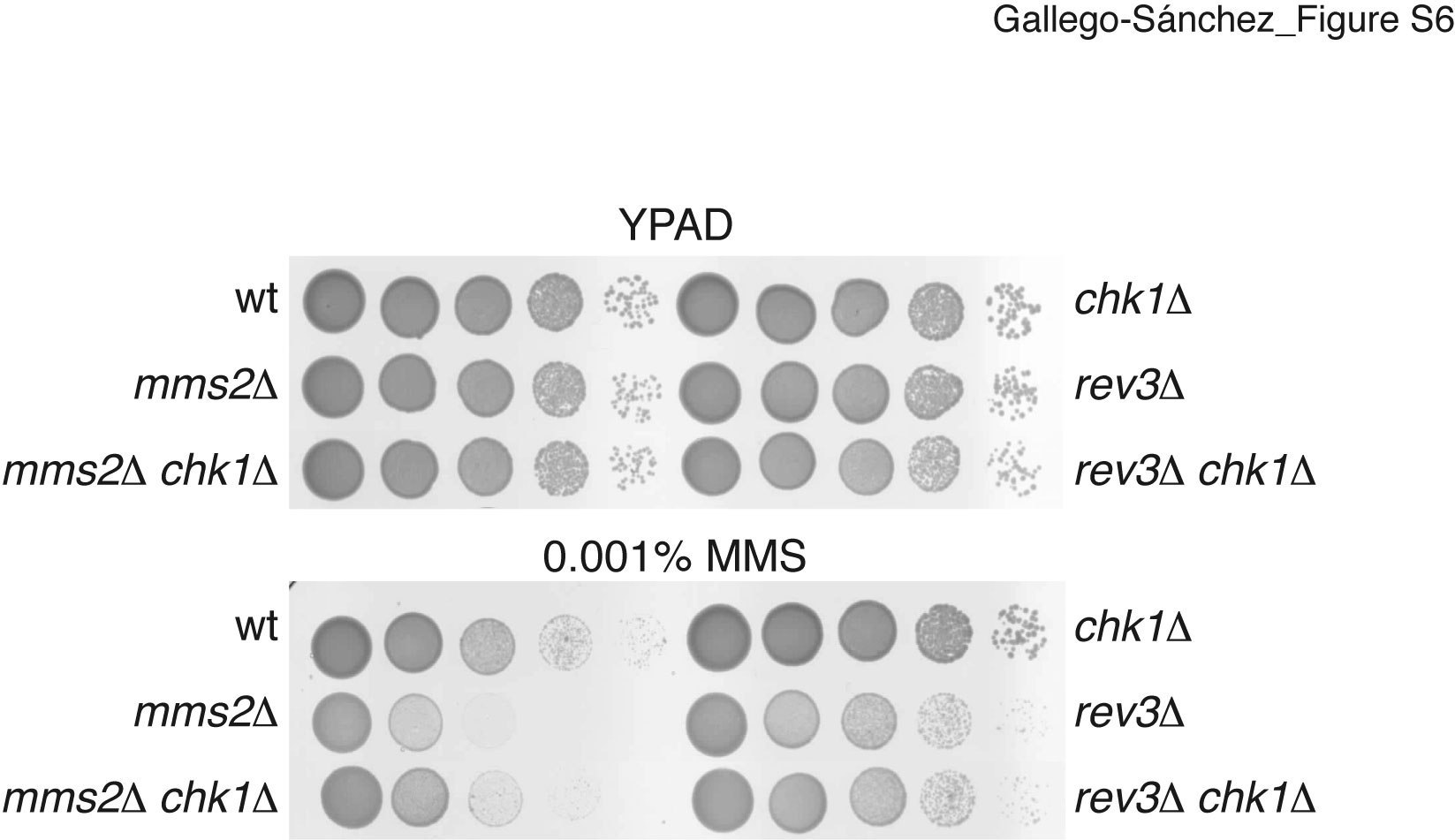

Supplement: Figure S6 — Analysis of the DNA damage tolerance pathway in a chk1 Δ chekpoint kinase mutant. Serial dilutions (ten-fold) of wild-type, chk1Δ, mms2Δ, rev3Δ, mms2Δ chk1Δ and rev3Δ chk1Δ strains plated on YPAD plates with MMS and without the alkylating chemical (as indicated). (JPG) [file pone.0081108.s006.jpg]

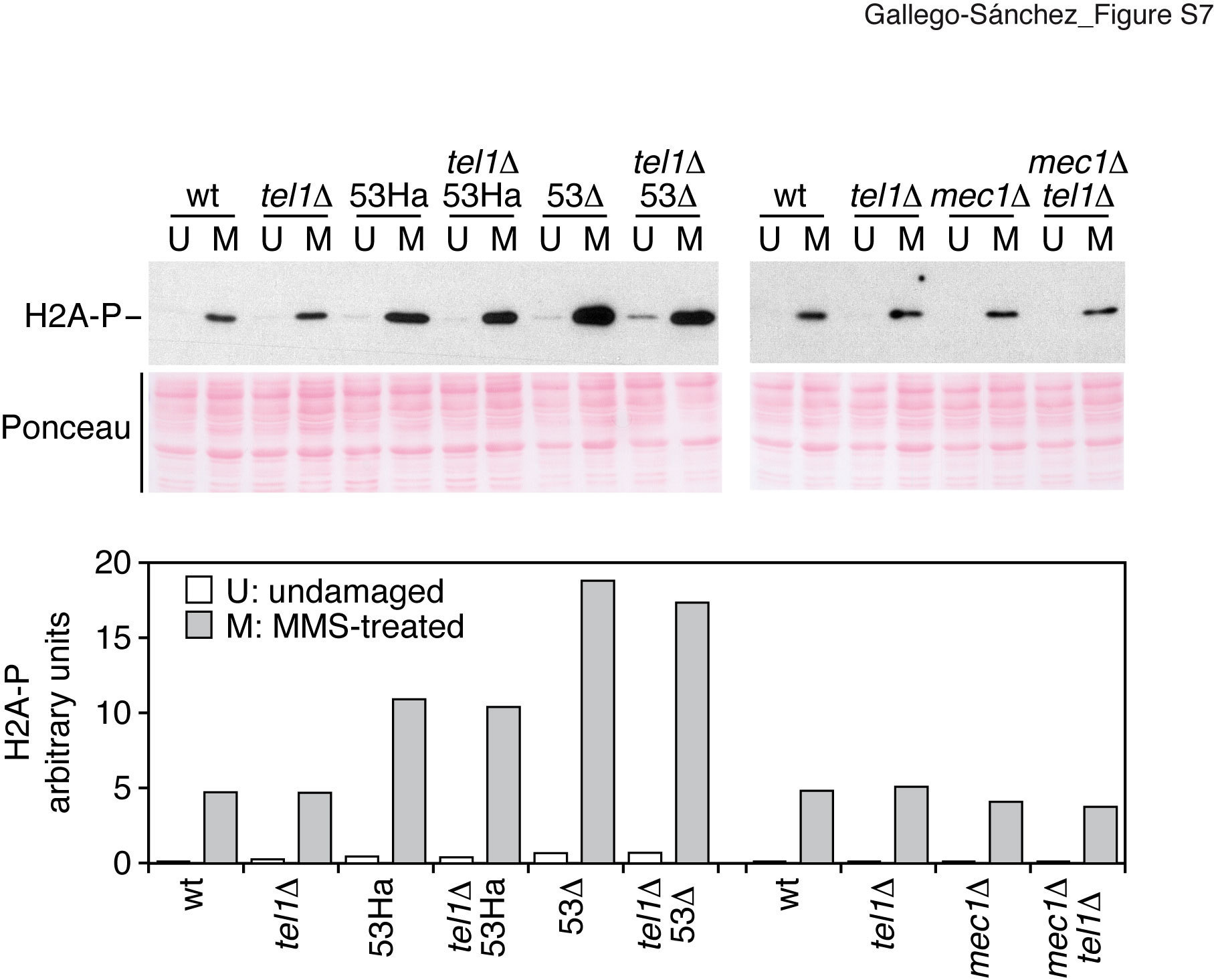

Supplement: Figure S7 — Phosphorylation of histone H2A in tel1 mutant cells. TCA-extracted protein samples of the indicated strans were taken from untreated cells (U) or 90 minutes MMS-treated cells (0.02% MMS except where indicated) (M), processed for Western blotting after SDS-PAGE in 13% gels and probed with α-γH2AX (ab15083, Abcam) to detect histone H2A phosphorylated at S129. A plot of the quantitation of H2A phosphorylation is shown. (JPG) [file pone.0081108.s007.jpg]

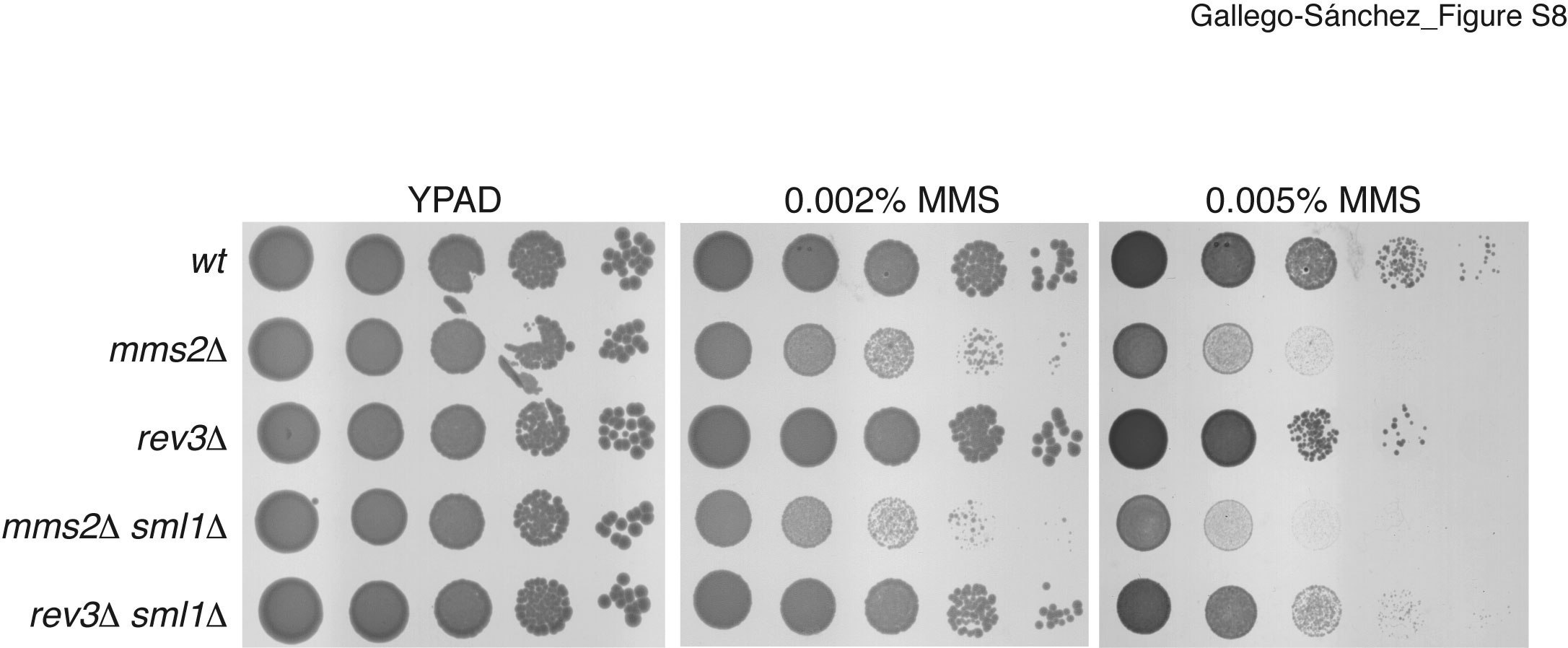

Supplement: Figure S8 — Analysis of the DNA damage tolerance pathway in mms2Δ sml1Δ and rev3Δ sml1Δ double mutants. Serial dilutions (ten-fold) of indicated strains plated on YPAD plates with MMS or without the alkylating chemical. The wild-type, mms2Δ, rev3Δ, mms2Δ sml1Δ and rev3Δ sml1Δ strains were assayed to test whether the sml1 mutation has any (additive) effect on mms2Δ or rev3Δ mutations. (JPG) [file pone.0081108.s008.jpg]

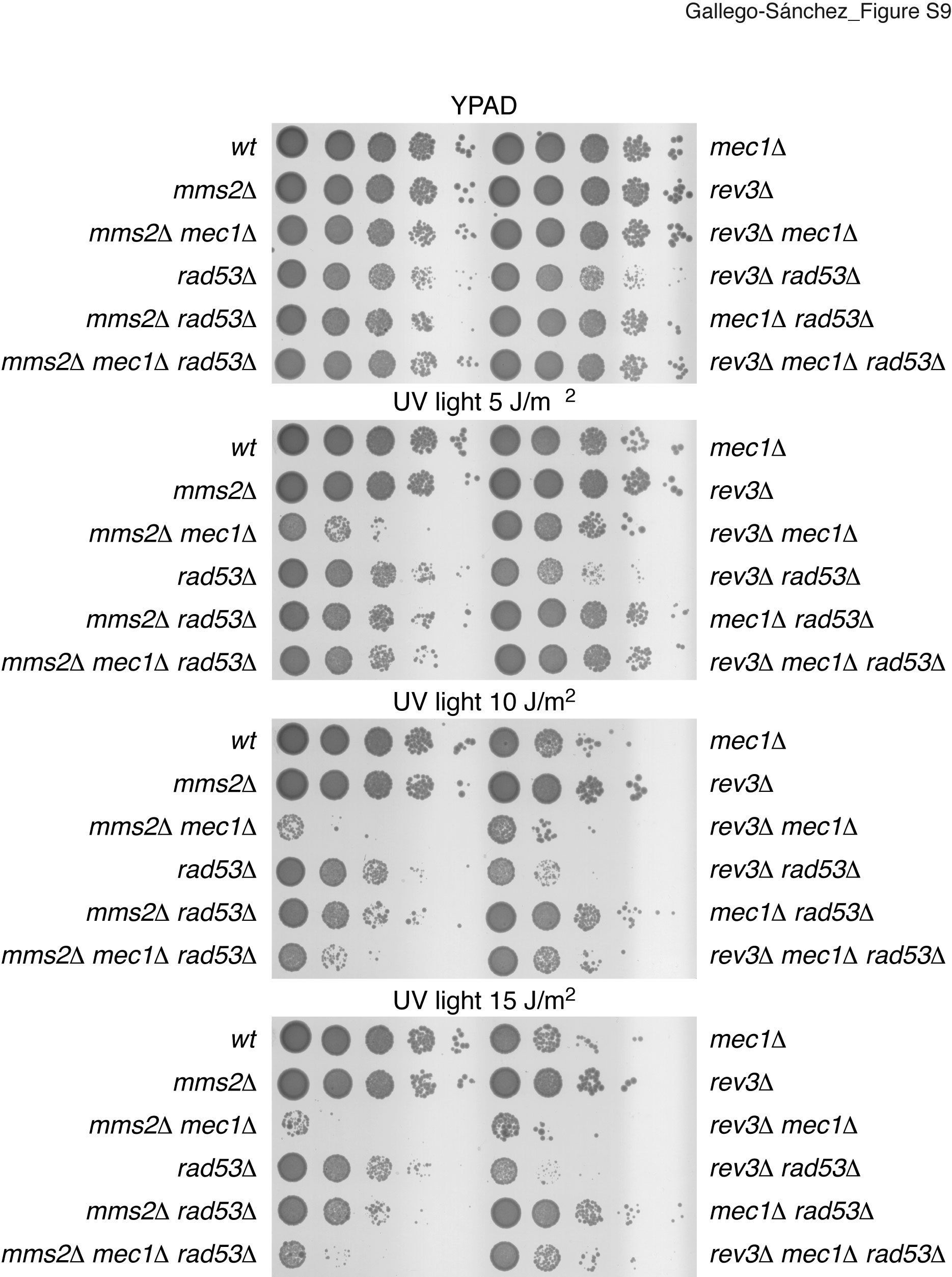

Supplement: Figure S9 — Analysis of the DNA damage tolerance pathway in mec1 Δ rad53 Δ double mutants. Serial dilutions (ten-fold) of indicated strains (see below) plated on YPAD plates and exposed to the indicated doses of UV radiation. wild-type, mec1Δ, mms2Δ, rev3Δ, mec1Δ mms2Δ, mec1Δ rev3Δ, rad53Δ, rad53Δ rev3Δ, rad53Δ mms2Δ, mec1Δ rad53Δ, mec1Δ rad53Δ mms2Δ and mec1Δ rad53Δ rev3Δ. Note that all the strains used in this assay were sml1Δ. (JPG) [file pone.0081108.s009.jpg]
