# Supplementary material for: Analysis of the Tolerance to DNA Alkylating Damage in MEC1 and RAD53 Checkpoint Mutants of Saccharomyces cerevisiae
Source: PLoS One. 2013 Nov 19;8(11):e81108. doi: 10.1371/journal.pone.0081108 (PMC3834268; doi:10.1371/journal.pone.0081108)
Supplement: Table S1 — List of strains used in this study. (PDF) [file pone.0081108.s010.pdf]

TABLE S1

Yeast strains used in this study. All strains are isogenic with the W303 background.

Strain	Genotype	Origin
55Sc34.08	*MAT**a** ade2-1 can1-100 his3-11 leu2,3-112 trp1-1 ura3-1 bar1Δ::LEU2*	G.C. Walker
56Sc37.08	55Sc34.08 with *rad53Ha:TRP1*	This work
57Sc74.08	55Sc34.08 with *mms2Δ::KanMX6*	This work
57Sc75.08	55Sc34.08 with *mms2Δ::KanMX6 rad53Ha:TRP1*	This work
55Sc35.08	55Sc34.08 with *rev1Δ::KanMX6*	GC Walker
55Sc36.08	55Sc34.08 with *rev3Δ::KanMX6*	GC Walker
55Sc37.08	55Sc34.08 with *rev7Δ::KanMX6*	GC Walker
55Sc70.08	55Sc34.08 with *rev1Δ::KanMX6 rad53Ha:TRP1*	This work
56Sc38.08	55Sc34.08 with *rev3Δ::KanMX6 rad53Ha:TRP1*	This work
56Sc39.08	55Sc34.08 with *rev7Δ::KanMX6 rad53Ha:TRP1*	This work
55Sc63.08	55Sc34.08 with $pol30^{K164R}$	This work
55Sc65.08	55Sc34.08 with $pol30^{K164R}$ *rad53Ha:TRP1*	This work
58Sc23.08	55Sc34.08 with *rad18Δ::KanMX6*	This work
58Sc24.08	55Sc34.08 with *rad18Δ::KanMX6 rad53Ha:TRP1*	This work
58Sc25.08	55Sc34.08 with *siz1Δ::KanMX6*	This work
58Sc26.08	55Sc34.08 with *siz1Δ::KanMX6 rad53Ha:TRP1*	This work
57Sc76.08	55Sc34.08 with *rev3Δ::KanMX6 mms2Δ::HIS3*	This work
57Sc77.08	55Sc34.08 with *rev3Δ::KanMX6 mms2Δ::HIS3 rad53Ha:TRP1*	This work
48Sc29.06	*MAT**a** ade2-1 can1-100 his3-11 leu2,3-112 trp1-1 ura3-1*	A. Bueno
48Sc32.06	48Sc29.06 with *rad53Ha:TRP1*	This work
SUO 1.56	48Sc29.06 with $rad53^{S350A,G404V}$*:KanMX6*	This work
57Sc52.08	48Sc29.06 with $rad53^{S350A,G404V}$*:KanMX6 mms2Δ::HIS3*	This work
57Sc53.08	48Sc29.06 with $rad53^{S350A,G404V}$*:KanMX6 rev3Δ::HIS3*	This work
48Sc37.08	48Sc29.06 with *slx4Δ::KanMX6*	This work
48Sc43.08	48Sc29.06 with *slx4Δ::KanMX6 rad53Ha:TRP1*	This work
54Sc32.08	48Sc29.06 with *mec1Δ::ADE2 sml1Δ::KanMX6*	This work
57Sc50.08	48Sc29.06 with *mec1Δ::ADE2 sml1Δ::KanMX6 mms2Δ::HIS3*	This work

57Sc51.08	48Sc29.06 with *mec1Δ::ADE2 sml1Δ::KanMX6 rev3Δ::HIS2*	This work
46Sc02.05	48Sc29.08 with *rad53Δ::HPH sml1Δ::TRP1*	This work
62.Sc.26.09	48Sc29.08 with *rad53Δ::HPH sml1Δ::TRP1 mms2Δ::KanMX6*	This work
62.Sc.27.09	48Sc29.08 with *rad53Δ::HPH sml1Δ::TRP1 rev3Δ::KanMX6*	This work
62.Sc.39.10	48Sc29.08 with *ddc1-2Ha:LEU2*	This work
62.Sc.40.10	48Sc29.08 with *mec1-1 ddc1-2Ha:LEU2*	This work
62.Sc.63.10	48Sc29.08 with *rad53Δ::HPH sml1Δ::TRP1 slx4-13myc:HIS3*	This work
62.Sc.64.10	48Sc29.08 with *mec1Δ::ADE2 sml1Δ::KanMX6 slx4-13myc:HIS3*	This work
62.Sc.65.10	48Sc29.08 with *rad53Ha:TRP1 ddc1-2Ha:LEU2*	This work
62.Sc.66.10	48Sc29.08 with *rad53Δ::HPH sml1Δ::TRP1 ddc1-2Ha:LEU2*	This work
65.Sc.23.10	48Sc29.06 with *mec1Δ::ADE2 rad53Δ::HPH sml1Δ::KanMX6*	This work
65.Sc.24.10	48Sc29.06 with *mec1Δ::ADE2 rad53Δ::HPH mms2Δ::HIS3 sml1Δ::KanMX6*	This work
65.Sc.25.10	48Sc29.06 with *mec1Δ::ADE2 rad53Δ::HPH rev3Δ::HIS3 sml1Δ::KanMX6*	This work
70.Sc.40.12	48Sc29.06 with *tel1Δ::KanMX6*	This work
70.Sc.42.12	48Sc32.06 with *tel1Δ::KanMX6*	This work
70.Sc.44.12	46Sc02.05 with *tel1Δ::KanMX6*	This work
70.Sc.46.12	54Sc32.08 with *tel1Δ::KanMX6*	This work
49Sc51.08	*MAT**a** ade2-1 can1-100 his3-11,15 his3-11 leu2,3-112 ura3-1 bar1Δ::URA3*	This work
49Sc52.08	49Sc51.08 with *rad53Ha:TRP1*	This work
54Sc10.08	49Sc51.08 with *mms2::KanMX6*	This work
54Sc11.08	49Sc51.08 with *mms2::KanMX6 rad53Ha:TRP1*	This work
54Sc12.08	49Sc51.08 with *rev3::KanMX6*	This work
54Sc13.08	49Sc51.08 with *rev3::KanMX6 rad53Ha:TRP1*	This work
54Sc14.08	49Sc51.08 with *esc4Δ::KanMX6*	This work
54Sc15.08	49Sc51.08 with *esc4Δ::KanMX6 rad53Ha:TRP1*	This work
SUO 2.04	49Sc51.08 with *chk1Δ::TRP1*	This work
55Sc49.08	49Sc51.08 with *chk1Δ::TRP1 mms2Δ::KanMX6*	This work
55Sc50.08	49Sc51.08 with *chk1Δ::TRP1 rev3Δ::KanMX6*	This work
